# Research Progress in Organic Photomultiplication Photodetectors

**DOI:** 10.3390/nano8090713

**Published:** 2018-09-11

**Authors:** Linlin Shi, Qiangbing Liang, Wenyan Wang, Ye Zhang, Guohui Li, Ting Ji, Yuying Hao, Yanxia Cui

**Affiliations:** 1 Key Laboratory of Advanced Transducers and Intelligent Control System of Ministry of Education, College of Physics and Optoelectronics, Taiyuan University of Technology, Taiyuan 030024, China; shilinlinsll@sina.cn (L.S.); liangqiangbing@126.com (Q.L.); wangwenyan@tyut.edu.cn (W.W.); zhangye@tyut.edu.cn (Y.Z.); liguohui@tyut.edu.cn (G.L.); jiting@tyut.edu.cn (T.J.); haoyuyinghyy@sina.com (Y.H.); 2 Key Laboratory of Interface Science and Engineering in Advanced Materials, Taiyuan University of Technology, Taiyuan 030024, China

**Keywords:** photodetector, organic, photomultiplication, tunneling, external quantum efficiency

## Abstract

Organic photomultiplication photodetectors have attracted considerable research interest due to their extremely high external quantum efficiency and corresponding high detectivity. Significant progress has been made in the aspects of their structural design and performance improvement in the past few years. There are two types of organic photomultiplication photodetectors, which are made of organic small molecular compounds and polymers. In this paper, the research progress in each type of organic photomultiplication photodetectors based on the trap assisted carrier tunneling effect is reviewed in detail. In addition, other mechanisms for the photomultiplication processes in organic devices are introduced. Finally, the paper is summarized and the prospects of future research into organic photomultiplication photodetectors are discussed.

## 1. Introduction

Photodetectors are optoelectronic devices which can absorb light energy and convert it into electrical energy, having found applications in wide areas of image sensing, missile guidance, environmental pollution monitoring, light communications, photometric metrology, industrial automation, and so on [1,2,3,4,5,6,7,8]. In particular applications, it is required that the sensitivity of photodetectors is sufficiently high to detect weak light signal, for example, bio-imaging sensing or long range light communication [9,10,11,12]. There are two routes to improve the sensitivity of photodetectors, one of which is to improve the external quantum efficiency (*EQE*) and the other is to reduce the dark current density. Using photomultiplication (PM) effect to improve the *EQE* is one of the most important approaches to achieve high-sensitivity photodetection.

Traditional photomultipliers, based on a complex vacuum system including photo induced electron emission, secondary electron emission, and electron optics possessing components, are bulky and of high cost, severely limiting their applications [13,14,15,16]. Avalanche photodiodes are another commonly used high-sensitivity photodetector, which are made of inorganic semiconductor materials such as silicon, germanium, indium gallium arsenide [13,17,18,19], and so on. Their working mechanism is that photo generated carriers are accelerated under the strong electric field induced by a large reverse bias, and then the impact ionization with the crystal lattice takes place, thereby bringing forward the avalanche multiplication effect [20,21,22].

Besides inorganic semiconductors, organic semiconductors have also been widely favored in the field of optoelectronics due to their advantages of a simple synthesized method and adjustable bandwidth, as well as their light weight, low cost, eco-friendliness, good flexibility, and so on. High performance organic photodetectors have been reported successively in the past few years [23,24,25,26,27], some of which also allow the excitation of PM effects, providing another route to realize high-sensitivity photodetectors [4,28,29,30,31,32]. Since the exciton binding energy of organic semiconductor materials is approximately 0.1–1.4 eV, about three orders higher than that of inorganic semiconductor materials, impact ionization cannot occur in organic PM photodetectors like in inorganic avalanche photodiodes. Instead, the working mechanism of organic PM photodetectors has been identified mainly due to the trap assisted carrier tunneling effects [33,34,35].

After more than 20 years of development, the active layer materials of organic PM photodetectors have transitioned from organic small molecular compounds to polymers, and their device performance has also been optimized constantly. In this review, we will firstly introduce the typical structures and working mechanisms of organic PM photodetectors along with their key performance parameters. Next, we will give a detailed review of the research progress for PM photodetectors based on organic small molecular compounds and polymers, respectively. Some important progresses in improving the quantum efficiency, dark current, response speed, and spectral performance of both types of PM photodetectors are presented. In addition, we will introduce some other working mechanisms of organic PM photodetectors. Finally, we will summarize the paper and consider prospects for the future research of organic PM photodetectors.

## 2. Basic Structures and Working Mechanisms of Organic PM Photodetectors

### 2.1. Basic Structures of Organic PM Photodetectors

**Figure 1:**
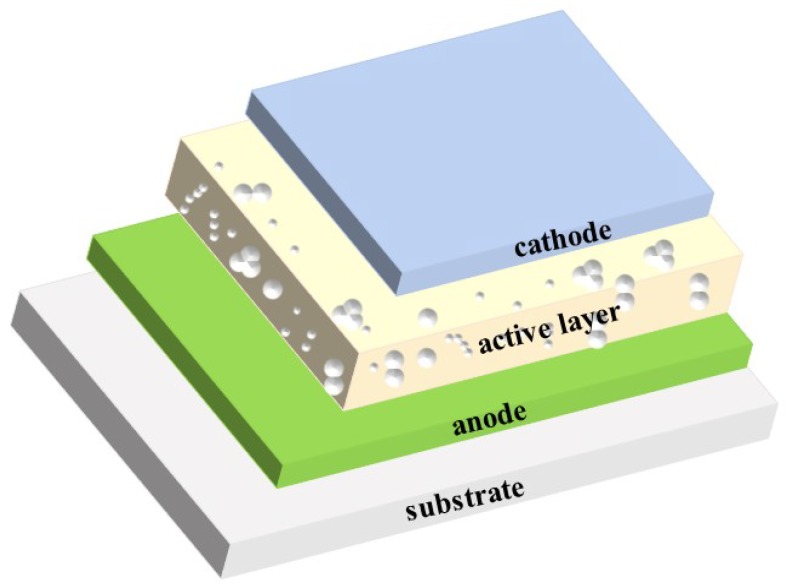
Structural diagram of organic PM photodetectors.

The basic structures of organic PM photodetectors as shown in Figure 1 comprises of the anode, the cathode, and the active layer with a large amount of interfacial/bulk carrier traps. They can be mainly grouped into two types, the single junction type and the bulk heterojunction type, which are similar to those of organic solar cells [36,37,38]. The early organic PM photodetectors belonged to the single junction type with their active layers made of an *N*-type or *P*-type organic compound. In contrast, the bulk heterojunction type organic PM photodetectors possess active layers made of donor/acceptor (D/A) blend. Although the bilayer heterojunction type organic solar cells, in which the active layer consists of a stack of *N*-type and *P*-type semiconductor films, have been frequently researched, there are rare studies about applying this junction in organic PM photodetectors. In practice, extensive efforts have been made on introducing interfacial modified layers between the electrode and the active layer to improve the PM effects in organic photodetectors. In addition, doping other materials into the active layer has also been carried out for improving PM performances.

### 2.2. Working Mechanisms of Organic PM Photodetectors

**Figure 2:**
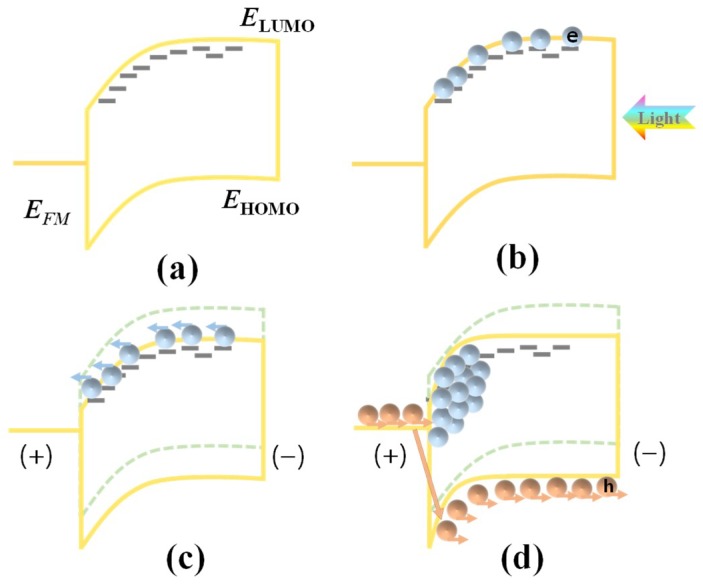
Working mechanism of organic photomultiplication (PM) photodetectors due to the electron trap assisted hole tunneling effect. (**a**) Energy band bending without bias; (**b**) Photo-generated electrons are captured by traps; (**c**) Trapped electrons transport toward the junction once the bias is applied; (**d**) Trapped electrons arriving at the junction cause the hole tunneling from the circuit into the semiconductor, producing the current multiplication effect. *E_FM_* is the Fermi level of the metal, and *E_LUMO_* and *E_HOMO_* are the lowest unoccupied molecular orbital level and the highest occupied molecular orbital level of the organic semiconductor, respectively.

Most of organic PM photodetectors realize *EQE* far exceeding 100% based on the trap assisted carrier tunneling effect. In all, the trap assisted carrier tunneling effect contains four steps which are the formation of the Schottky barrier, the capture of carriers by traps after light illuminations, the carrier transport toward the Schottky junction under applied bias, and the carrier tunneling through the narrowed Schottky barrier. Based on the type of the trapped carrier (electron or hole), the working mechanisms of organic PM photodetectors are divided into two categories. Figure 2 shows the working mechanism of the case of electron trap assisted carrier tunneling effect.

The premise of realizing the carrier tunneling effect is the formation of Schottky junction when the metal electrode is in contact with the semiconductor layer [39]. For the organic semiconductor with a large number of electron traps, the proper Schottky band bending without bias is shown in Figure 2a. It corresponds to the case when the Fermi energy level of the metal electrode (*E_FM_*) is higher than that of the organic semiconductor material (*E_FS_*). In such a case, electrons flow from metal into the semiconductor, yielding a built-in electric field toward the semiconductor with a downward energy band bending, thereby hindering the diffusion of holes in the organic semiconductor to the electrode. To achieve such a downward band bending, low work function electrodes like Ag (4.26 eV), Al (4.28 eV), and Mg (3.66 eV), are required. After light illumination, photo-generated electrons are captured by the electron traps as shown in Figure 2b. When a bias is applied with the electric field pointing from electrode to the semiconductor (reverse bias if the electrode works as cathode; forward bias if the electrode works as anode), the trapped electrons transport toward the Schottky junction as shown in Figure 2c. In the junction region, these arrived electrons narrow the Schottky junction and thus enhance the intensity of the built-in electric field, causing the holes to tunnel through the junction and inject into the organic semiconductor from the external circuit, and finally resulting in the current multiplication effect, as shown in Figure 2d.

The corresponding processes of hole trap assisted electron tunneling effect are exactly the opposite (not shown), which requires *E_FM_* lower than *E_FS_*. Using a high work function metal such as Au (5.1 eV), ITO (4.7 eV), Pt (5.65 eV), and so on, can fulfill such a condition, thereby an upward band bending is constructed in the semiconductor. In addition, the applied bias for trapped hole transportation is the reverse, with the electric field pointing from the semiconductor to the electrode, facilitating the holes transport toward the Schottky junction. In practice, the surface of the metal electrode is always modified in order to adjust its work function and further regulate the band bending.

### 2.3. Key Performance Parameters of Organic PM Photodetectors

Key performance parameters of organic PM photodetectors include photoresponsivity, quantum efficiency, detectivity, linear dynamic range, and response time, which are listed as follows.

#### 2.3.1. Photoresponsivity

The photoresponsivity is defined as the ratio of photocurrent to the power intensity of incident light, which characterizes the sensitivity of photodetectors to incident light. The greater photoresponsivity, the better sensitivity to incident light for PM photodetectors. Photoresponsivity can be expressed by Equation (1):

(1) $$R=\frac{I_{ph}}{P_{in}}=\frac{I_{l}-I_{d}}{P_{in}}$$

where *I_ph_* is photocurrent, *I_l_* is the current under light illumination, *I_d_* is dark current, and *P_in_* is the incident light intensity.

#### 2.3.2. External (Internal) Quantum Efficiency

The external quantum efficiency (*EQE*) is defined as the electron number detected per incident photon, as presented by Equation (2):

(2) $$EQE=\frac{N_{e}}{N_{p}}=\frac{I_{ph}/e}{P_{in}/h\nu }$$

where *N_e_* and *N_p_* are the number of detected electrons and incident photons, respectively, *h* is the Planck’s constant, *ν* is the frequency of light, and *e* is the electronic charge. The absorption properties of the selected materials, the structural design of the device, and the electrical properties of the materials are all key factors affecting *EQE*. In traditional photodiode type photodetectors, *EQE* is smaller than unity. However, if current gain exists, *EQE* can be greater than 1, for example, in avalanche photodiode, photoconductor, or phototransistor type photodetectors [40,41].

In organic photomultiplication photodetectors, the presence of deep traps in the organic active layer causes a long carrier recombination lifetime for one type of charge, resulting in a high photocurrent amplification (gain), similar to that happens in photoconductor type photodetectors. Gain is determined by the ratio of recombination lifetime and transit time for another type of charge to sweep across the device, as given by Equation (3):

(3) $$Gain=\frac{\chi \tau }{T}=\frac{\chi \tau \mu V}{L^{2}}$$

where *χ* is the fraction of trapped electrons or holes over the total amount of the dissociated excitons, *τ* is the lifetime of trapped carriers, *T* is the transport time of the untrapped carriers flowing across the active layers, *V* is the applied bias, *L* is the active layer thickness, and *μ* is the field dependent mobility of the untrapped carriers. As known, in photodetectors with gain mechanisms, *EQE* is equal to *Gain* in number [42].

The internal quantum efficiency (*IQE*) is defined by the ratio of the number of carriers detected in the external circuit to the number of photons absorbed. The product of the *IQE* and the light absorption efficiency of the active layer (*abs*) is the *EQE*, as determined by Equation (4):

(4) $$IQE=\frac{N_{e}}{N_{p}abs(\lambda )}=\frac{EQE}{abs(\lambda )}$$

#### 2.3.3. Detectivity

The detectivity (D*) is the figure of merit to characterize the capability of weak light detection for a photodetector, which can be calculated from the noise density and the photoresponsivity *R*. It is one of the most important physical parameter for photodetectors as given by Equation (5):

(5) $$D^{*}=\frac{R\sqrt{Af}}{i_{n}}$$

where *A* is the active area of the detector, *f* is the electrical bandwidth, and *i_n_* is the measured total noise current. Considering that the noise current under dark is dominated by shot noise, the detectivity can be calculated through Equation (6):

(6) $$D^{*}=\frac{R}{\sqrt{2eJ_{d}}}$$

#### 2.3.4. Linear Dynamic Range

The linear dynamic range (*LDR*) is defined as the response range of photodetector being linear over a wide range of light intensity. *LDR* can be calculated through Equation (7):

(7) $$LDR=20\log(\frac{P_{\max}}{P_{\min}})$$

where and *P_min_* are the maximum and minimum incident light intensities of the photocurrent density versus light intensity curve which lie within the linear response range.

#### 2.3.5. Response Time

The response time of the detector reflect the response speed of the detector to receive incident light radiation, which includes two parts, the rise time (*T_r_*) and the falling time (*T_f_*). The rise (or falling) time is defined as the time for the photocurrent to rise from 10% to 90% (fall from 90% to 10%) during the on and off cycles of light illumination. The sum of the rise time and the falling time is counted as the response time of the photodetector.

## 3. Organic PM Photodetector Based on Small Molecular Compounds

The early organic PM photodetectors were based on organic small molecular compounds such as *N*-methyl-3,4,9,10-perylenetetracarboxyl-diimide (Me-PTC), naphthalene tetracarboxylic anhydride (NTCDA), fullerenes (C_60_), 2,9-dimethyl quinacridone (DQ), and so on. Except for DQ, Me-PTC, NTCDA, and C_60_ are all *N*-type semiconductor materials which support the hole trap assisted electron tunneling effect. In this section, we will first introduce the progress of organic small molecular PM photodetectors based on the single junction type and the bulk heterojunction type, respectively. Then, some important progress made by researchers in improving the performance of the organic small molecular PM photodetectors will be presented.

### 3.1. Single Junction Type Organic Small Molecular PM Photodetectors

**Figure 3:**
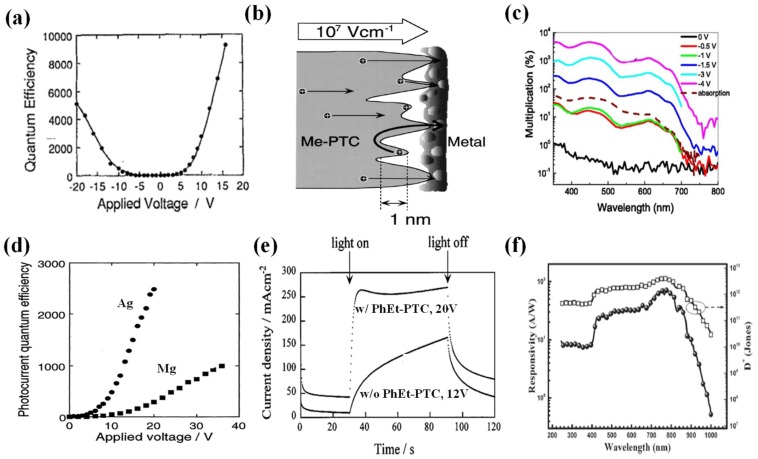
Device performances of various organic small molecular PM photodetectors. (**a**) Internal quantum efficiency (*IQE*) at different voltages for the Au/Me-PTC/Au device under 600 nm light illumination (Reproduced with permission from [43]. *AIP Publishing*, 1994); (**b**) Schematic view of the interfacial traps for the Au/Me-PTC/ITO device (Reproduced with permission from [44]. *AIP Publishing*, 1998); (**c**) External quantum efficiency (*EQE*) spectra under different biases and the absorption spectrum of the ITO/PEDOT:PSS/C_60_/BCP/Al device (Reproduced with permission from [49]. *AIP Publishing*, 2007); (**d**) *IQE* at different voltages under 600 nm light illumination for the ITO/DQ/Ag and ITO/DQ/Mg devices, respectively (Reproduced with permission from [50]. *The Japan Society of Applied Physics*, 1996); (**e**) Transient current density curves of ITO/PhEt-PTC/NTCDA/Au and ITO/NTCDA/Au devices, respectively (Reproduced with permission from [26]. *AIP Publishing*, 2000); (**f**) Responsivity and detectivity spectra of the glass/ITO/TPBi/C_70_/SnPc:C_70_/BCP/Al incorporated with down-conversion material of 4P-NPB (Reproduced with permission from [28]. *Royal Society of Chemistry*, 2016).

In 1994, Hiramoto et al. fabricated the pioneering organic PM photodetector using the Me-PTC (a *N*-type perylene pigment having methyl groups) based on a triple layer configuration of Au/Me-PTC/Au on a glass substrate. The fabricated device produced an *IQE* of 1.0 × 10^4^ at −16 V under 600 nm light illumination at the temperature of −50 °C, as shown in Figure 3a [43]. Their later experimental work reflected that the surface of Me-PTC was very rough, leading to imperfect contact between and the metal and Me-PTC and thereby forming structural traps of holes, as indicated in Figure 3b [44]. As a result, a large number of holes were trapped by Me-PTC at the metal/Me-PTC interface, making the PM phenomenon possible. However, Me-PTC based organic PM devices cannot respond at room temperature due to too few interfacial trapped carriers. Besides Me-PTC, two other perylene pigments of PhEt-PTC and n-Bu-PTC also exhibit the PM effect due to structural traps at the imperfect metal/semiconductor interface [45,46]. Later, the same group realized the PM photodetection at room temperature based on organic small molecular material of NTCDA. The NTCDA device presented a PM effect under 400 nm light illumination and its *IQE* reached 1.3 × 10^5^ at −16 V [47]. Subsequent studies indicate that introducing an interfacial layer of PhEt-PTC next to the NTCDA layer [26] or reducing the grain boundaries of the NTCDA film [48] can improve the response speed of the device (see the detail in Section 3.3.4).

The organic small molecular material C_60_ is also a commonly used material for achieving PM effects. In 2007, Huang and Yang characterized the photo current response of a device with configuration of ITO/PEDOT:PSS/C_60_/BCP/Al, in which ITO/PEDOT:PSS was used to form a composite electrode [49]. Here, C_60_ layer formed a disordered structure, which is better than ordered structures for producing the PM effect. With the help of the composite electrode, they realize an *EQE* of 5.0 × 10^3^% as shown in Figure 3c based on the hole trap assisted electron tunneling effect. By increasing the bias voltage the PM effect becomes more significant. However, inserting a 20 nm thick BCP layer at the PEDOT:PSS/C_60_ interface would annihilate the PM performance, reflecting that the PM behavior occurs at the PEDOT:PSS/C_60_ interface rather than within the C_60_ layer.

In contrast, the organic PM devices based on the electron trap assisted hole tunneling effect have been researched not as extensively as their hole trap counterparts. In 1996, Hiramoto et al. developed the first electron trap based PM photodetector using a *P*-type material Quinacridone (DQ) as the active layer [50]. In their device, the DQ layer was sandwiched between ITO and Ag (or Mg) electrodes. This study concluded the metal electrode plays a significant role in influencing the multiplication performance of the photodetectors. The Ag electrode with a work function a bit higher than that of the Mg electrode is much superior on producing a high multiplication factor. Specifically, the Ag electrode device has an *IQE* of 2.5 × 10^3^ when the bias voltage is 20 V while that of the Mg electrode device is only 1.0 × 10^3^ at a bias of 36 V; see in Figure 3d.

### 3.2. Bulk Heterojunction Type Organic Small Molecular PM Photodetectors

The organic small molecular PM photodetectors with bulk heterojunction type active layers were proposed later than their single junction counterparts. So far, all reported bulk heterojunction type organic small molecular PM photodetectors are made of fullerene and a *P*-type semiconductor material through co-evaporation. As early as 2002, Matsunobu et al. firstly put forward the ITO/CuPc:C_60_/Au device for PM photodetection [51]. They found that the rise and fall times of the bulk heterojunction device are only 8 ms and 15 ms, respectively, while the response time of the single junction control device is in second time scale. This is mainly because the introduction of CuPc provides a favorable path for hole transport, therefore the accumulation time of holes at the Schottky junction can be shortened.

In 2010, Hammond et al. prepared a bulk heterojunction type PM photodetector with a structure of ITO/NTCDA/C_60_/CuPc:C_60_/BCP/Al [52]. They found that the insertion of a composite hole blocking layer comprising of NTCDA (2–3 nm)/C_60_ (10 nm) can facilitate the accumulation of holes at the interface between the hole blocking layer and the active layer, inducing a large amount of electrons being injected from the electrode and thereby producing a high photocurrent gain. When the composite hole blocking layer is removed, the device *EQE* increases at 0 V bias, but reduces to be less than 100% as the applied voltage increases. Such a phenomenon proves that the gain of the device occurs at the interface between the hole blocking layer and the photoactive layer, rather than within the photoactive layer.

Recently, bulk heterojunction organic small molecular PM photodetectors with a configuration of ITO/TPBi (or Bmpypb, LiF)/C_70_/TAPC (or SnPc):C_70_/BCP/Al were developed [28]. Here, the co-evaporated film of TAPC (or SnPc):C_70_ works as the active layer. They further elucidated that for the composite interfacial layer of TPBi (or Bmpypb, LiF)/C_70_, TPBi (or Bmpypb, LiF) plays the role of hole blocking while C_70_ plays the role of hole accumulation. The *EQE* of their TAPC:C_70_ PM devices exceed 1.0 × 10^4^% over the wavelength range from 350 nm to 650 nm and the response of the SnPc:C_70_ device can be extended to infrared (IR) range.

### 3.3. Performance Studies on Organic Small Molecular PM Photodetectors

In this subsection, we will introduce the progress of performance studies on organic small molecular PM photodetectors from the aspects of water/oxygen treatment, quantum efficiency, dark current, response speed, and spectrum adjustment.

#### 3.3.1. Water/Oxygen Treatment

Because organic materials are sensitive to water and oxygen, organic semiconductor films and devices are usually prepared in conditions without water and oxygen. However, studies reflect that performances of the prepared organic PM photodetectors bear dramatic changes after being exposed to water and oxygen [53,54]. Sometimes, the water or oxygen treatment is beneficial to the improvement of the photo current gain. For example, Hiramoto et al. found that the adsorption of oxygen by *N*-type materials of Me-PTC and NTCDA suppresses the PM effect while the same treatment toward *P*-type material of DQ increases the PM gain factor. This is because there is more O_2_^−^ after the organic material adsorbing O_2_ molecules. The increased O_2_^−^ act as electron traps which facilitate the carrier capture in *P*-type semiconductor and thereby more holes being injected, but inhibit the carrier capture in *N*-type semiconductor due to recombination with hole traps. They also found that after the adsorption of water, devices made of *N*-type material Me-PTC and *P*-type material DQ exhibited higher PM performance due to the increased photocarrier generation.

#### 3.3.2. Quantum Efficiency

Devices with higher quantum efficiency have higher responsivity and the corresponding devices are more sensitive at fixed dark current. The early study showed that the hole traps within Me-PTC based PM devices are structural traps produced by non-uniform Me-PTC film interfaces. From this point, research on the effect of deposition rate and deposition method of the metal electrode on the PM performance was carried out [55]. That work concluded that, based on the thermal evaporation method, decreasing the deposition rate of metal electrode from 0.7 nm/s to 0.008 nm/s brings forward the increase of the quantum efficiency by 30 times. This is because the low rate of deposition of the Au electrode can maintain the structural traps produced by the non-uniform Me-PTC film. In addition, they found that the ion sputtering method is less effective than the thermal evaporation method for yielding high PM performance due to the same reason. For the PM photodetector made of the *N*-type perylene pigments, its quantum efficiency can also be improved through the solvent treatment [45]. It was demonstrated that the THF solvent treatment can change the PhEt-PTC film from amorphous to poly-crystalline, therefore the PhEt-PTC becomes coarser, producing more structural traps and thus elevating the quantum efficiency, as exhibited in Figure 3d.

#### 3.3.3. Dark Current

The dark current determines the ability of detecting weak light for photodetectors. When the quantum efficiency of different devices are comparable, the device with lower dark current has lower noise equivalent power, which allows a weaker optical signal to be detected. Jinsong Huang and his collaborators found that the dark current of PM photodetector with configuration of ITO/PEDOT:PSS/C_60_/BCP/Al was as high as 2 mA/cm^2^ at −6 V, not suitable for the detection of weak light. They attributed the large dark current to the possible ohmic contact between PEDOT:PSS and C_60_. In order to reduce dark current, a high carrier injection barrier is required. The same group found that by inserting a C-TPD layer between the PEDOT:PSS and C_60_ films, an electron injection barrier as high as 2.8 eV was realized, thereby significantly reducing the dark current by 3–4 orders [56]. But such a design brings barrier for electron injection from PEDOT:PSS to C_60_, resulting in the *EQE* lower than 100%. Later, they introduced a nano-composite buffer layer C-TPD:ZnO (1:1) between PEDOT:PSS and C_60_, through which they not only maintained the dark current at a low level but also realized the PM photodetection [57]. Compared with the device with a single C-TPD buffer layer, the composite buffer layer with ZnO nanoparticles brought additional hole traps, which could capture photo generated holes, making them recombine with the electrons in the defect state on the ZnO surface. As a result, the energy band bending between ZnO nanoparticles can be reduced, promoting the electron injection from PEDOT:PSS to C_60_, and therefore the PM effect can be realized. Due to the reduced dark current, its LDR of PM photodetection reaches 120 dB and its detectivity at 390 nm is as high as 3.6 × 10^11^ Jones.

#### 3.3.4. Response Speed

Photodetectors with high response speed are crucial for many applications. Increasing the applied bias can shorten the response time. Usually, a high quality active layer can withstand a high applied voltage with respect to a poor quality one. In 2000, Nakayama et al. compared the performances of ITO/PhEt-PTC/NTCDA/Au and ITO/NTCDA/Au devices [26]. In their experiments, they failed to fabricate high quality NTCDA film but succeeded in making high quality PhEt-PTC film on ITO. With the help of high quality PhEt-PTC buffer film, the morphology of the NTCDA film can be improved apparently. As a result, the PhEt-PTC/NTCDA based device can withstand a high voltage. The transient measurement displays that the rise time of the PhEt-PTC/NTCDA device is only 3.7 s at a bias voltage of −20 V while that of the control is more than 60 s at −12 V, as exhibited in Figure 3e. It is emphasized that the insertion of a PhEt-PTC layer does not have negative effects on the electron injection from ITO to NTCDA because the LUMO level of PhEt-PTC is lower than that of the NTCDA. In addition, the response speed of the photodetector can be faster using a single-crystalline semiconductor, due to good carrier transport property, with respect to the polycrystalline one [48].

#### 3.3.5. Spectrum Adjustment

Another important indicator of photodetector performances is its working wavelength range. Generally, a narrow band photodetector can be realized through a broadband photodetector integrated with a color filter. Thus, a high sensitivity photodetector that can respond at wavelength ranges of ultraviolet (UV), visible, and even IR would be quite attractive because of its wide applications.

The combination of two active materials is one approach to realize broadband response of PM devices. For example, NTCDA based PM devices can only sense UV light due to its large band gap, but in combination with Me-PTC, the composite device can respond to both UV light and the visible light [47]. High multiplication rate at the visible range is produced by the photo carrier generation taking place within the Me-PTC layer. It is noted that the triple layer device of ITO/Me-PTC/Au can only work at temperatures below zero Celsius. However, the bilayer system of NTCDA/Me-PTC allows the generation of holes by light absorption in Me-PTC and trapping of holes in the NTCDA film, resulting in the accumulation of holes at the NTCDA/Au interface and further the injection of electrons into the device.

Light transition based on down conversion materials is another effective way to broaden the spectral range of PM devices. Recently, aiming to enhance the response at the deep-UV range, Yang et al. applied a capping layer with the down conversion material of 4P-NPB on the illumination side of a bulk heterojunction PM device with configuration of glass/ITO/TPBi/C_70_/SnPc:C_70_/BCP/Al [28]. Their work demonstrates that when the UV light irradiates the 4P-NPB layer, the device will absorb the light and emit visible light, which will pass through into SnPc:C_70_ layer and then produce the PM response. Under illumination by light that can be absorbed by SnPc:C_70_, the 4P-NPB layer is transparent, yielding negligible influences on the multiplication rates. Overall, the wavelength range of the PM device with detectivity exceeding 10^11^ Jones covers from deep-UV to near-IR (250–1000 nm) as shown in the Figure 3f, and the responsivity and detectivity at 780 nm are 70 A/W and 4 × 10^12^ Jones, respectively.

## 4. Organic PM Photodetectors Based on Polymers

With respect to the small molecular counterparts, although polymer PM photodetectors were developed later, they have attracted significant interests and attention of researchers attributed to their advantages of rich materials, easy process, and good compatibility with the roll-to-roll technique. Studies on single junction type polymer PM photodetectors are scarce. In 1999, Däubler et al. fabricated a single junction type polymer PM photodetector based on a *P*-type semiconductor material arylamino-PPV. Due to the large amount of electron accumulation at the arylamino-PPV/Al interface, the tunneling of holes from Al into the active layer takes place, yielding the PM photodetection with an *IQE* up to 2.0 × 10^3^% [58]. In 2007, Campbell and Crone also observed PM in a device with configuration of ITO/PEDOT:PSS/MEH-PPV/Al [59] and its gain is around 20 under 500 nm light illumination at −20 V, but they provided an explanation that is distinct from the trapped carrier induced carrier injection.

In the following years, almost all related studies have focused on bulk heterojunction type polymer PM photodetectors, which can be categorized into three groups, depending on the heterojunction objects. The most widely studied group is the bulk heterojunction formed by organic semiconductors (e.g., polymer and fullerene derivative), and the other two are heterojunctions with inorganic materials and insulating polymers. In this section, we will first introduce the progress made in these three heterojunction type polymer PM photodetectors, respectively. Next, performance studies including reducing dark current and broad/narrow band spectrum adjustment of polymer PM photodetectors will be disclosed.

### 4.1. Bulk Heterojunction Based on Organic Semiconductors

We will introduce the progress made in bulk heterojunction polymer photodetectors based on organic semiconductors according to different donor/acceptor weight ratios.

#### 4.1.1. Donor/Acceptor Weight Ratio of 1:1

In the early days, by reference to solar cells, bulk heterojunction type polymer PM devices were designed with the donor/acceptor weight ratio of 1:1. Experiences suggest that 1:1 donor/acceptor weight ratio forms a favorable interpenetrating network in solar cells which can facilitate the transport of both electrons and holes. However, with such design, one can hardly achieve current multiplication because of the short life time of both electrons and holes. To realize PM photodetection in a P3HT:PC_61_BM (with the weight ratio of 1:1) device, one can incorporate inorganic nanoparticles into the active layer which will be presented in Section 4.2 [65]. Other approaches of doping organic compounds [60,66] and interface modifications [61,67,68] have been also proposed to realize the PM phenomenon in 1:1 donor/acceptor heterojunction devices as introduced in the following.

**Figure 4:**
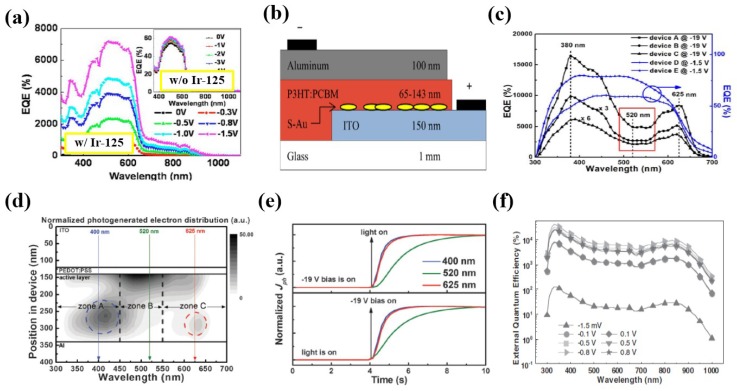
Device performances of various polymer PM photodetectors based on bulk heterojunctions made of organic semiconductors. (**a**) *EQE* spectra of ITO/PEDOT:PSS/P3HT:PCBM (1:1)/Ca/Al with and without Ir-125 doped in active layer (Reproduced with permission from [60]. *AIP Publishing*, 2010); (**b**) Structural diagram of ITO/s-Au/P3HT:PCBM/Al device (Reproduced with permission from [61]. *AIP Publishing*, 2014); (**c**) *EQE* spectra of ITO/PEDOT:PSS/P3HT:PC71BM/LiF/Al with different P3HT:PC71BM weight ratios (Reproduced with permission from [62]. *Springer Nature*, 2015); (**d**) Calculated wavelength dependent distribution of photogenerated electrons in the active layers of P3HT:PC71BM (100:1) without bias (Reproduced with permission from [63]. *Royal Society of Chemistry*, 2015); (**e**) Normalized transient photo current curves under light illumination at the wavelengths of 400 nm, 520 nm, and 625 nm, respectively for P3HT:PC71BM (100:1) device (Reproduced with permission from [63]. *Royal Society of Chemistry*, 2015); (**f**) *EQE* spectra measured under different bias voltages after UV light treatment for ITO/ZnO/PDPP3T:PC71BM (1:2)/Al device (Reproduced with permission from [64]. *John Wiley and Sons*, 2016).

In 2010, Chen et al. incorporated an organic dye Ir-125 into the P3HT:PC_61_BM (1:1) bulk heterojunction device and discovered current multiplication as well [60]. The device without dye does not have any gain. In contrast, the device with dye show PM from UV to near IR range as shown in Figure 4a. The *EQE* reaches its maximum of 7.2 × 10^3^% at −1.5 V under 500 nm light illumination. The exhibited PM phenomenon is because Ir-125 dye brings forward a lot of electron traps into the active layer. With the incorporation of another organic dye Q-switch 1, the PM response of the P3HT:PC_61_BM:Ir-125 device can be extended to near IR range [66].

Besides, interface modification is an alternative effective approach to induce PM in P3HT:PC_61_BM (1:1) devices. In 2014, Melancon et al. introduced a semi-continuous gold (s-Au) film between the ITO electrode and the active layer, with its structural diagram displayed in Figure 4b. Their work indicated that with the help of the s-Au film, PM was successfully excited with an *EQE* of 1.5 × 10^3^% at −2 V bias under 400 nm light illumination [61]. They explained this phenomenon that the s-Au film acts as a hole blocking layer which enables the accumulation of holes at the P3HT/PCBM interface and the further tunneling of electrons from ITO into the P3HT/PCBM region. In 2017, Wang et al. used PFN to modify the ITO/active layer interface but the obtained *EQE* was only slightly higher than 100% [67]. Later, the same group proposed to utilize the transparent polyethylenimine ethoxylated (PEIE) to modify the ITO surface [67]. Through this process, the work function of the electrode gets lower, bringing forward the energy barrier formed between the work function of the PEIE modified ITO and the HOMO of P3HT is 0.75 eV larger than that between the bare ITO and P3HT. The expanded energy barrier causes the enhanced interfacial accumulation of photo carrier, which is preferred to the increased photocurrent gain. The *EQE* value of the device based on PEIE modified ITO reached 3.3 × 10^3^% at −1 V under 370 nm light illumination. The proposed PM device exhibits a rise time of 78 μs and a fall time of 87 μs.

#### 4.1.2. Donor/Acceptor Weight Ratio Higher than 1:1

An easy means to realize PM in the donor/acceptor heterojunction photodetectors is by increasing the amount of donor (or reducing the content of acceptor) in the blend of active material, in other words, using a blend with donor/acceptor weight ration higher than 1:1. On the condition that the ratio of acceptor in the active layer is being reduced, the acceptor forms isolated islands instead of connected networks, which can trap the photo generated electrons and thereby making possible the injection of holes from the external circuit. Compared with PM devices with balanced donor/acceptor weights, the response speed of bulk heterojunction polymer PM photodetectors with less acceptor in the active layer is significantly lower, because it takes more time for trapped carriers to accumulate at the Schottky junction due to poorer carrier transport properties.

The pioneer research of this kind of PM devices was carried out by Fujun Zhang’s group in 2015 [62]. They fabricated a PM photodetector with a configuration of ITO/PEDOT:PSS/P3HT:PC_71_BM/LiF/Al using 100:1 P3HT/PC_71_BM which exhibited an *EQE* as high as 1.7 × 10^4^% under 380 nm light irradiation at −19 V bias, as shown in Figure 4c. The acceptor islands can trap the photo generated electrons, which will transport to and accumulate at the Schottky junction formed between the active layer and the Al electrode with applied voltage. Later, by removing the LiF buffer layer, they lowered the hole injection barrier between the active layer and the Al electrode, raising the *EQE* up to 3.8 × 10^4^% at −19 V bias [69]. P3HT molecular arrangement with face-on is more favorable for hole transportation. Their further study indicated that rapid annealing the active layer after spin-coating can avoid atomic self-assembly, which is more helpful to form a face-on structure, bringing forward *EQE* rise up to 1.2 × 10^5^% at −19 V under 610 nm light illumination due to the improved hole transport property [70]. Subsequently, they employed a burn-in treatment to their PM devices by applying a voltage of −25 V and −19 V for 1000 s, successively, making the device performance more stable [63]. In order to further elucidate the working mechanism of the devices, they examined the distributions of optical field within in the PM device at different wavelengths together with the transient photocurrent measurements; see Figure 4d,e. The results indicated that stronger absorption occurring closer to the Al electrode (e.g., at wavelengths of 400 nm and 625 nm) corresponds to a faster transient response due to shorter distance of electron transport. In contrast, at 520 nm wavelength light illumination, although with relatively high light absorption, its transient response is quite slow due to the absorption taking place close to the PEDOT:PSS/P3HT:PC_71_BM interface. Besides PCBM, they also used IC_60_BA to make the active layer with P3HT but the obtained *EQE* was 6.9 × 10^2^% with a 100:2 donor/acceptor weight ratio [71].

Most of the active layers in bulk heterojunction polymer PM devices comprise of polymer and fullerene derivative of PCBM. Beyond fullerene and their derivatives, non-fullerene acceptor materials have also attracted attention due to their strong absorption capabilities in visible and near-IR regions, adjustable energy levels, and good stability. Through collaboration with Xiaowei Zhan’s group, Zhang’s group prepared the PM devices by blending P3HT with non-fullerene acceptor materials of DC-IDT2T [72] or ITIC [73], resulting in extended spectrum response and simultaneously more stable device performances. For the DC-IDT2T based PM device, its optimal donor/acceptor weight ratio was 100:1 as well and its *EQE* exceeded 1.0 × 10^4^% over the range from 350 nm to 650 nm with a maximum *R* of 131.4 A/W and D* of 1.43 × 10^14^ Jones. Moreover, compared with the PCBM based device, the DC-IDT2T based device responded much better at the near-IR wavelength range due to good absorption of DC-IDT2T. After exposing the PM devices in air for 40 h, the DC-IDT2T based device only suffered 39% degradation on *EQE* while the PCBM based device bore a degradation of 57%, indicating the DC-IDT2T acceptors are promising for developing stable PM photodetectors.

The *P*-type polymer constituting the active layer of PM photodetectors can also be regulated. In 2017, Esopi et al. used a *P*-type material F8T2 as the donor to prepare the PM devices with PCBM [74]. Their device configuration is ITO/PEDOT:PSS/F8T2:PC_71_BM (100:4)/LiF/Al with the F8T2/PC_71_BM weight ratio of 100:4 and its *EQE* is 5.6 × 10^3^% at −40 V bias under 360 nm light illumination. Most importantly, F8T2 based PM devices have a very low dark current (only 2.7 × 10^−7^ mA/cm^2^ at −1 V bias), much lower than that of the P3HT based device. This is due to the inhibited hole injection from the Al electrode to the donor, produced by the increased barrier between the HOMO level of the acceptor and Fermi level of Al (F8T2: 1.2 eV, and P3HT: 0.9 eV). But the increased barrier inevitably brought the decrease of photodetector response under light illumination. They also compared the PM performances before and after removing the LiF buffer layer, and concluded that the removal of LiF made the hole injection much easier but it also deteriorated the stability of devices.

#### 4.1.3. Donor/Acceptor Weight Ratio Lower than 1:1

It is anticipated that decreasing the donor/acceptor weight ratio would increase the amount of hole traps in the active layer, possibly leading to PM photodetection based on the hole trap assisted electron tunneling effect. In 2016, Dongge Ma and collaborators proposed a PM photodetector based on a narrow bandgap polymer donor PDPDP3T with the configuration of ITO/ZnO/PDPP3T:PC_71_BM/Al, which showed an *EQE* of 1.4 × 10^5^% at a low bias of −0.5 V after the device was irradiated by UV light for 30 s, as displayed in Figure 4e [64]. In their active layer, the blend of PDPP3T:PC_71_BM system has a weight ratio of 1:2 (lower than 1:1), which is different from the previous two situations in Section 4.1.1 and Section 4.1.2. Here, they claimed that there are a lot of hole traps in their active layer, which tended to form trapped holes accumulated at the ZnO/active layer interface under reverse bias. Because large electron injection barriers were generated at both the ITO/ZnO and ZnO/active layer interfaces, the pristine device without UV treatment behaves as a photodiode without any gain. The electron blocking effect could be alleviated through UV treatment. After absorbing ultraviolet light, the ZnO nanoparticles could generate electron-hole pairs, functioning as centers to neutralize the oxygen molecules adsorbed on the surface of the ZnO nanoparticles. As a result of the desorption of oxygen molecules from the surface of the particles, a decrease of the LUMO energy differences at both the ITO/ZnO and ZnO/active layer interfaces took place, and a reduction of the electron injection barriers from ITO to PC_71_BM was realized. Such a phenomenon finally enabled the device to become a photoconductor with large gain.

Differently, the investigation carried out by Nie et al. into an inverted organic PM photodetector of ITO/lysine/PBDTT-DPP:PC_71_BM (1:2)/MoO_3_/Al also included an active layer with less donor. In 2017, electron traps were identified rather than hole traps [75]. In this design, the ITO modified by lysine was the cathode while the MoO_3_/Al acted as the anode. The device showed a large amount of photon to electron multiplication at room temperature with an *EQE* up to 1.6 × 10^5^% (936.05 A/W) under 10 V bias. Inversely, no gain was found at negative bias. The gain behavior was attributed to the electron trap assisted hole tunneling from Al/MoO_3_ composite electrode into the active layer. The low current under dark was due to a space charge region formed between PBDTT-DPP/MoO_3_ interfaces, which could be erased after being exposed to light.

### 4.2. Bulk Heterojunction with Inorganic Nanoparticles or Quantum Dots

Both inorganic nanoparticles and quantum dots have been employed to form heterojunctions with polymer or polymer blend. Their incorporation provides additional carrier traps which are essential for the followed carrier tunneling at the Schottky junction.

In 2008, Chen et al. incorporated cadmium telluride (CdTe) nanoparticles into the active layer of P3HT:PC_61_BM (1:1), obtaining an *EQE* of 8.0 × 10^3^% at −4.5 V bias under 350 nm light illumination [65]. In this study, their CdTe nanoparticles were capped with *N*-phenyl-*N*-methylthiocarbamate (PMDTC) ligands which can improve the solubility of inorganic nanoparticles in the target solution of active material. Their study implied that a solvent annealing step after film spin-coating could induce a higher concentration of CdTe nanoparticles on the top of the annealed film. Under light exposure, CdTe nanoparticles with trapped electrons lowered the energy barrier for hole injection from the top electrode to the active layer.

**Figure 5:**
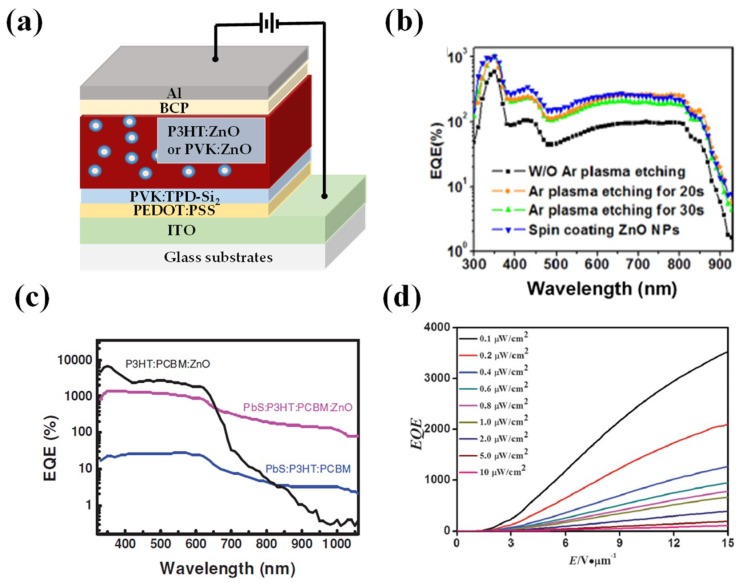
Device performances of various polymer PM photodetectors based on bulk heterojunctions with inorganic materials and insulating polymers. (**a**) Structural diagram of ITO/PEDOT:PSS/PVK:TPD-Si_2_/P3HT:ZnO (PVK:ZnO)/BCP/Al device; (**b**) *EQE* spectra of the PDTP-DFBT based PM photodetectors blended with ZnO nanoparticles treated by different processes (Reproduced with permission from [76]. *AIP Publishing* 2015); (**c**) *EQE* spectra of the ITO/PEDOT:PSS/P3HT:PCBM/Al devices with the active layer doped with or without PbS and ZnO QDs (Reproduced with permission from [77]. *John Wiley and Sons*, 2014); (**d**) *EQE* spectra under different light intensities of the bulk heterojunction polymer PM photodetector realized through doping Y-TiOPc quantum dots into the insulating polymer PVB (Reproduced with permission from [27]. *Royal Society of Chemistry*, 2016).

ZnO nanoparticles are an alternative choice of carrier trap materials which have the merits of low cost, variable synthetic strategies, and so on. In 2012, Jinsong Huang’s group incorporated ZnO nanoparticles into P3HT or PVK film and fabricated the PM devices with configurations of ITO/PEDOT:PSS/PVK:TPD-Si_2_/P3HT (or PVK):ZnO/BCP/Al as shown in Figure 5a [8], aiming to modify the Schottky junction for smooth hole injection into the active layer. Here, the PVK:TPD-Si_2_ blend film behaved as the electron blocking layer and the BCP layer acted as the hole blocking layer, through which the dark current was controlled at an extremely low level (6.8 nA at −9 V for the PVK based device) while excellent PM performance was maintained. Specifically, the *EQE* of the P3HT:ZnO device and PVK:ZnO device are up to 13.4 × 10^5^% and 2.4 × 10^5^% respectively, at −9 V bias under 360 nm light illumination. Similar to the function of CdTe nanoparticles in the previous work, ZnO nanoparticles blended with the donor polymer worked as the electron traps which could modify the Schottky junction for smooth hole injection into the active layer. Later, Huang’s group incorporated ZnO nanoparticles into PDTP-DFBT film which can sense light from UV to near-IR [76]. They demonstrated that the surface treatment of active layer by Ar plasma etching can effectively enhance electron trap assisted hole injection with the gain improved by 2–3 times as displayed in Figure 5b. A control sample of spin-coating ZnO nanoparticle layer on the top of active layer provided a direct evidence that excess ZnO nanoparticles created more traps.

Beyond nanoparticles, inorganic quantum dots with sizes smaller than 10 nm have also been applied in organic PM photodetectors aiming for providing carrier traps or extending the response spectrum range. In 2014, Huang’s group doped PbS quantum dots and ZnO quantum dots together into the active layer comprising of P3HT:PC_61_BM (1:1) [77]. The ternary active layer of P3HT:PC_61_BM:ZnO already possesses the PM photodetection ability at wavelengths shorter than 650 nm because ZnO quantum dots offer plentiful electron traps and thus enable hole tunneling. With the incorporation of PbS quantum dots, the PM performance was extended to the wavelength as long as 1000 nm, as shown in Figure 5c. The principle is that the electrons generated due to light absorption by PbS in the IR range can transfer to ZnO traps, triggering the hole injection into the active layer and further PM photodetection with extended spectrum range.

### 4.3. Bulk Heterojunction with Insulating Polymers

It is an interesting finding that blending organic quantum dots into insulating polymer can also induce PM photodetection. In 2015, Peng et al. proposed a bulk heterojunction photodetector made of oxotitanium phthalocyanine in the crystal form of phase-Y (Y-TiOPc) quantum dots and an insulating polycarbonate resin PCZ-300 (shorted as Y-TiOPc@PC) in contact with two parallel metal electrodes, which had a wide spectral response from 400 nm to 940 nm and an *EQE* of 3.6 × 10^4^% at 830 nm [78]. The explanation of their finding is as follows. In the device, Y-TiOPc nanoparticles were separated by PC, forming a large amount of Y-TiOPc/PC interfaces, therefore the only allowed charge transport mechanism in the active layer is based on charge tunneling. Because the energy barrier for the hole is higher by 1.4 eV than that for the electron, the tunneling probability of photo generated electrons in the conduction band is estimated to be much higher than that of photo-induced holes in the valence band of the separated Y-TiOPc quantum dots. As a result, efficient trapping of holes is produced, so that more electrons can flow through the device before the recombination of photo induced charge carriers occurs. Actually, this work did not mention the tunneling of carrier at the interface between the active layer and electrode, thus its principle should be different from that presented in Section 2.2 We will come back to this kind of mechanism for organic PM photodetection in Section 5.

Subsequently, Li et al. proposed a polymer PM photodetector based on Y-TiOPc quantum dots as well [27]. In that work, the Y-TiOPc quantum dots were dispersed into a polyvinyl butyral (PVB) based solution to make the active layer, and a blended film of polycarbonate (PC) and m-TPD were selected as the hole transport layer. The prepared photodetector exhibited an obvious PM phenomenon with the highest *EQE* of 3.5 × 10^5^% (see in Figure 5d), an excellent photosensitivity with the maximum responsivity of 2227 A/W, and an outstanding low-light detection with the highest normalized detectivity of 3.1 × 10^14^ Jones under 780 nm light illumination. Different from the explanation in previous work [78], they attributed the multiplication to the enhanced external hole tunneling injection assisted by trapped electrons at the interface of active layer and ITO. They found that the hole energy barrier was only 0.2 eV at the Y-TiOPc/m-TPD interface while the electron energy barrier reached 0.7 eV at the Y-TiOPc/ITO interface, causing an unbalanced transport of electrons and holes. Such an unbalance further lead to the accumulation of electrons at the Y-TiOPc/ITO interface, enabling the following narrowing of Schottky junction and thus hole tunneling. Their study also reflected that Y-TiOPc quantum dots with smaller diameter could not only generate more photo carriers, but also contribute to the formation of a steeper band bending, promoting the injection of a large amount of holes.

### 4.4. Performance Studies of Polymer PM Photodetectors

In this subsection, we will introduce the progress of performance studies on polymer PM photodetectors from the aspects of dark current, broadband response, and narrowband response.

#### 4.4.1. Dark Current

**Figure 6:**
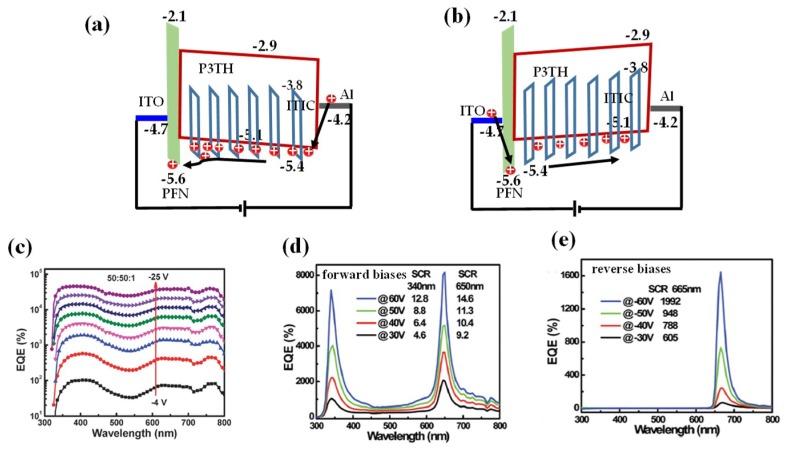
(**a**,**b**) Energy level diagrams of the PFN device under reverse forward biases under dark; (**c**) *EQE* spectra under different biases of the ITO/PEDOT:PSS/P3HT:PTB7-Th:PC_71_BM (50:50:1)/Al device (Reproduced with permission from [79]. *Royal Society of Chemistry*, 2015); (**d**,**e**) Narrowband *EQE* spectra of the ITO/PFN-OX/P3HT:PC_61_BM (4.0 μm)/Al device under different biases (Reproduced with permission from [92]. *Royal Society of Chemistry*, 2017).

Inserting an appropriate buffer layer between the active layer and electrode can greatly reduce the dark current while maintaining the photo current [67,73]. For example, Zhang’s group systemically compared the performances of ITIC based polymer PM photodetectors using PEDOT:PSS and PFN as the buffer layer with their current density-voltage (*J*-*V*) characteristics under dark and light respectively [73]. It was found that the PEDOT:PSS device can only work under reverse bias; in contrast, the PFN device can work effectively under both reverse and forward bias. Moreover, the dark current of the PFN device (10^−6^ mA/cm^2^) was much lower with respect to the PEDOT:PSS device (10^−4^ mA/cm^2^) at 0 bias; the dark current of the PFN device also decreased significantly at the bias when PM was triggered (e.g., −15 V). Such a phenomenon was explained as follows based on the energy diagrams displayed in Figure 6a,b. Under the reverse bias, the amount of holes injected from the Al electrode to the HOMO level of P3HT is relatively low because the difference between the LUMO level of P3HT and the Fermi level of Al is high (about 1.2 eV), causing the dark currents in both PEDOT:PSS and PFN devices to be low. The difference in performance between PFN and PEDOT:PSS devices is mainly due to difference between HOMO levels of these two buffer materials (PEDOT:PSS: −5.1 eV; PFN: −5.6 eV). Therefore, PFN behaves more effectively than PEDOT:PSS to prevent holes transit from active layer to the ITO electrode, yielding a reduced dark current. Under forward bias, the high HOMO level of PEDOT:PSS cannot block the hole injection from ITO to the active layer under dark, resulting in no response difference before and after light illumination. In contrast, there is a difference of 0.9 eV between the HOMO level of PFN and the Fermi level of ITO, which can effectively block holes being injected from the ITO electrode, yielding a very low dark current under forward bias.

#### 4.4.2. Broadband Response

In order to extend the response spectrum range of organic PM devices, researcher constructed ternary bulk heterojunctions which are realized through blending two donors with different absorption spectrum range together with the acceptor, similar as in solar cells [79,80,81,82,83,84,85]. In 2015, Zhang’s group prepared an polymer PM photodetector with the structure of ITO/PEDOT:PSS/P3HT:PTB7-Th:PC_71_BM/Al [79]. The photodetector with the active layer containing only PTB7-Th possess very weak PM effect, while the ternary device shows very high multiplication rates over the wavelength range from 350 nm to 800 nm. The highest *EQE* values of the ternary device with P3HT:PTB7-Th:PC_71_BM weight ratio of 50:50:1 are around 3.8 × 10^4^% in the spectral range from 625 nm to 750 nm under −25 V bias (as shown in Figure 6c), and the corresponding *R* is 229.5 A/W and D* is 1.91 × 10^13^ Jones. The broad spectral response range was due to the contribution of PTB7-Th exciton dissociation on the number of trapped electrons in PC_71_BM near the Al cathode. The results also showed that, with respect to P3HT/PC_71_BM junctions, PTB7-Th/PC_71_BM junction induced shallower traps due to smaller LUMO differences between donor and acceptor, resulting in quick filling of the electrons as turning on the incident light and thereby a faster response.

#### 4.4.3. Narrowband Response

Traditional photodetectors obtain narrowband response utilizing color filters, which are at the cost of light attenuation, the reduced responsivity, as well as the relatively complicated system with the increased cost [86,87,88,89,90]. In 2015, the Paul L. Burn and Paul Meredith’s group firstly achieved narrowband organic photodetector with thick active layers through adjusting the carrier collection efficiency [88]. Later, Zhang’s group applied the exact idea into polymer PM devices and implemented a filterless narrowband PM photodetector. The thicknesses of their active layers (100:1 P3HT:PC_71_BM) were in micrometer scale and the highest *EQE* reached 5.3 × 10^4^% at −60 V and the FWHM of their detection spectrum was only 28 nm [91]. Its principle is that, with thick active layer, close to the Al electrode, the amount of photo carriers decreases linearly from short wavelength to long wavelength, leading to a wavelength regime with poor hole collection efficiency at the opposite electrode under reverse bias. By using a PFN type buffer layer, the narrowband PM photodetector can work under both forward and reverse bias [92], with the reasons being present in Section 4.4.1. The PFN based PM device exhibited two narrowband response windows under forward bias (*EQE*: 7.2 × 10^3^% or 8.2 × 10^3^%, for 340 nm or 650 nm light illumination at 60 V) and a single narrowband response window under reverse bias (*EQE*: 1.6 × 10^3^% for 665 nm light illumination at −60 V) as displayed in Figure 6d,e, respectively. The two narrowband response windows obtained under forward bias can be well explained based on the photo carrier generation maps as functions of position and wavelength. Close to the ITO/PFN electrode, the photo carriers in the wavelength range of 400–600 nm is much larger, corresponding to poorer hole collection efficiency at the Al electrode due to long transport length, than that outside this range. As a result, under forward bias, two wavelength regimes are left with good hole collection efficiency, that is, the bands are shorter than 400 nm and longer than 600 nm. In 2018, they also applied this idea into ternary PM photodetectors [93]. Thanks to the doped PTB7-Th, the device had a narrowband response under −50 V reverse bias, corresponding to a maximum *EQE* of 2.0 × 10^2^% at 800 nm with an FWHM of 40 nm.

Similar works have also been carried out for bulk heterojunction polymer PM photodetectors based on inorganic quantum dots. In 2016, Huang’s group developed a narrowband PM photodetector with the structure of ITO/PVK/P3HT:PC_60_BM:CdTe QDs/BCP/Al with a 3.5 μm thick active layer thickness [94]. Compared with the quantum dot free photodetector, the photodetector with CdTe quantum dots maintained the low dark current under reverse bias as well as the narrow band response. Besides, the *EQE* values of the photodetector were improved significantly with the incorporation of the quantum dots which provide vast electron traps in the active layer, reaching nearly 2.0 × 10^2^% at 660 nm light irradiation and the corresponding LDR is 110 dB. Through a device with configuration of ITO/SnO_2_/PEIE/PDTP-DFBT:PC_71_BM:PbS QDs (4 μm-thick)/MoO_3_/Ag [95], they also obtained a filterless narrowband photodetection response with a 50 nm FWHM at near-IR range. In their design, PbS QDs behaved as hole traps, triggering the injection of electrons from Ag electrode to the active layer.

## 5. Other Mechanisms of Organic PM Photodetectors

In addition to the explanations presented in Section 2.2, researchers have also put forward some other mechanisms for realizing organic PM photodetection.

In 2006, Reynaert et al. used the organic small molecular F_16_CuPc as the active layer to prepare an organic PM photodetector with a structure of ITO/PEDOT:PSS/F_16_CuPc/Al, yielding the *EQE* exceeding 3.0 × 10^3^% at 633 nm [96]. They believed that the PM effect of this device was caused by the local charge induced exciton quenching in semiconductors. The reason is that F_16_CuPc is a unipolar disordered organic small molecular that forms an ohmic contact with the metal electrode rather than a Schottky contact. To verify the deduction, they compared the device performances with different metal electrodes. When the Schottky contact is formed at the semiconductor/metal interface, band bending will be different for metal electrodes with different work functions, thereby the device performances would be altered. However, their experiments indicated that the devices with Au electrodes and Al electrodes have exactly the same performance, reflecting that the ohmic contact is constructed between F_16_CuPc and the metal electrode. This theory is distinct from the trap assisted carrier tunneling mechanism, because the PM phenomenon of this F_16_CuPc based device is a bulk effect rather than an interfacial effect.

There are also other works confirming that the gain of their organic PM detectors is not due to interfacial effects. In 2007, Campbell and Crone prepared a series of organic PM photodetectors using MEH-PPV as the active layer [59]. They found the device with bare MEH:PPV had similar gain characteristics as those of devices with active layers doped with 10 wt% PbSe quantum dots or 10 wt% C_60_. Without mentioning any interfacial effects, they proposed that a small fraction of the optically excited excitons dissociate producing deeply trapped electrons and free holes. The trapped electrons can lead to photoconductive gain if the electron lifetime is longer than the hole carrier transit time. The gain per trapped electron is determined by the number of holes passing through the device during the lifetime of the trapped electron. This principle is similar to that of photoconductive type photodetectors. Later in 2009, the same researchers realized PM photodetection at near-IR range through a device with configuration of ITO/PEDOT/OSnNcCl_2_/BCP/Ca [97] based on the circulation of free carriers in response to trapped photo carriers as well. Peng et al. clarified that their Y-TiOPc@PC based PM devices were based on the same principle of carrier circulation [78].

One can also employ inorganic quantum dots to realize PM photodetection based on the principle of multiple exciton generation. In 2005, Qi et al. prepared an organic PM photodetector by doping PbSe QDs in MEH-PPV with the structure of ITO/PEDOT:PSS/PbSe:MEH-PPV/Al [98]. According to their study, when MEH-PPV was doped with PbSe quantum dots (with an absorption peak at 1900 nm) with a diameter of 8 nm, the device’s *EQE* reaches 1.5 × 10^2^% at −8 V bias under 510 nm light illumination. In contrast, the reference device only showed an *EQE* of 40%. Their explanation was as follows. When the incident photon energy was at least three times larger than the quantum dot band gap, the PbSe QDs could absorb photons and generate multiple excitons, resulting in multiplication of carriers. Another control study was carried out by altering the size of the PbSe quantum dots to 4.5 nm which corresponds to an absorption peak at 1100 nm, but the device did not induce any PM effect. The dependence of this photodetector performance on the size of quantum dots reflects that multiple exciton generation ascribed to the strong quantum confinement effect of quantum dots is responsible for the PM effect.

## 6. Summary and Outlook 

In this paper, we have summarized past studies on organic PM photodetectors since 1994. Performances of representative organic small molecular PM photodetectors and polymer PM photodetectors are summarized in Table 1 and Table 2, respectively.

Studies on organic small molecular PM photodetectors came out early. From Table 1, one sees clearly that most of reported studies focused on *N*-type semiconductor materials which produced PM performances based on the mechanisms of hole trap assisted electron tunneling. In these devices, the carriers are captured by interfacial structural traps, which bare the limitations of low quantity and complexity to control. In addition, single junction type PM devices suffer slow transient response, that is in second time scale or even longer, due to poor carrier transport in the active layer. These limitations were alleviated by constructing a bulk heterojunction active layer. C_60_ (or C_70_) was frequently combined with *P*-type semiconductor materials (e.g., CuPc, TAPC, SnPc) to form bulk heterojunction active layers, based on which the response time of PM photodetection can be reduced to millisecond timescale. The reason of improved response speed is that the trapped hole carriers can freely transport through the network formed by the *P*-type semiconductor material and thus the accumulation of carrier at the Schottky junction can be accelerated.

Table 2 reflects that polymer PM photodetectors have become a hot research topic since 2010, which must be closely related with the success made in the field of polymer solar cells. Compared with the single junction counterparts which have received little attention, bulk heterojunction type polymer PM photodetectors with diverse heterojunction formulas have been widely studied. For the heterojunction formula of polymer/inorganic-nanoparticles, Huang et al. realized an *EQE* of 3.4 × 10^5^% by blending ZnO nanoparticles into the active layer P3HT [8]. For the heterojunction formula of polymer/insulator, Li et al. proposed to make the active layer by dispersing the Y-TiOPc quantum dots into a PVB solution [27], yielding an *EQE* of 3.5 × 10^5^%. Beyond these two heterojunction formulas, the most popular researched one is the formula made of organic semiconductors comprising of donor and acceptor with different weight ratios. Introducing carrier traps is the primary task to realize current multiplication in organic bulk heterojunction type photodetectors. In 2015, Zhang et al. for the first time put forward introducing electron traps in the donor/acceptor active layer through reducing the weight ratio of acceptor, yielding an *EQE* of 1.7 × 10^4^% [62]. To bring in carrier traps into bulk heterojunction photodetector devices with 1:1 donor/acceptor weight ratio, approaches of doping organic dyes or incorporation of a gold island film have been adopted, but the achieved *EQE*s are far below those obtained by reducing the acceptor ratio. We also noticed that using PDPP3T:PC_71_BM blend with a weight ratio of 1:2 can also induce a PM effect with an *EQE* as high as 1.4 × 10^5^% after the LUMO energy differences at both the ITO/ZnO and ZnO/active layer interfaces are reduced by UV irradiation [64].

By comparison with organic small molecules which can only be processed through film deposition techniques, it is concluded that the solution processable polymers are the best candidates for developing low cost organic PM photodetectors because the solution process technique allows introduction of carrier traps into the bulk heterojunction active layer through blending. We also note that among the diverse methods of introducing traps into the polymer bulk heterojunction active layer, adjusting the weight ratio of acceptor/donor is the simplest one and researches on this topic might achieve greet success in the future. In order to improve the multiplication rate, it was necessary to insert a composite layer comprising of a hole blocking layer and a hole accumulation layer between the bulk heterojunction active layer and the electrode. The selection of the inserted hole blocking layer needs to be carried out delicately. On one hand, its HOMO level should be sufficiently lower than that of the *P*-type material in bulk heterojunction for reducing dark current under dark; on the other hand, its LUMO level should not be higher than that of *N*-type material in bulk heterojunction, enabling fluent electron injection from the electrode to the active layer. In addition, organic PM devices with broadband response, which can be sensitive to the light over wavelength ranges of ultraviolet (UV), visible, and even IR, can be realized through assembly of two different active materials as well as incorporation of down conversion materials, organic dyes, and so on. Especially, for polymer PM photodetectors, Zhang et al. have constructed a series of ternary bulk heterojunctions through blending two donors which respond at different spectrum ranges together with the acceptor [79,80] In order to realize filterless narrowband organic PM photodetectors, active layers with thicknesses of several micrometers were utilized to adjust the carrier collection efficiency.

Beyond the exotic achievements listed in this review for organic PM photodetectors, there are still some problems to be solved. For example, the response speeds of most reported organic PM photodetectors are slow, which cannot meet the needs of high speed photodetectors. In most of the work, the stabilities of the organic PM devices are not discussed, which is crucial to solve before putting them into practical applications. Moreover, the introduction of metal micro/nano structures into organic PM photodetectors should be consolidated with more attention as a feasible approach of manipulating the carrier generation and distribution like in organic solar cells [99,100,101,102,103,104,105,106,107,108,109,110,111]. It is emphasized that, recently, organic-inorganic hybrid perovskite photodetectors have also been reported with extremely high quantum efficiency [112,113,114,115] of which the principles were explained based on the trapped carrier assisted carrier tunneling or ion migration effect. And compared with organic devices, perovskite PM photodetectors require lower bias and possess faster response but their stability is inferior. Furthermore, it’s worth noting that, there are also plenty of original works on organic/inorganic hybrid heterojunction photodetectors with the inorganic materials of diverse structures including bulk films [116,117,118], 2D materials [119,120,121], nanomaterials [121,122], etc. A combination of organic materials with perovskite or inorganic semiconductors might offer a promising route to realize overall high performance PM photodetectors.

## Figures and Tables

**Table 1:** Representative organic small molecular PM photodetectors and their performances.

Mechanism	SJ/BJ	Year [Ref]	Device Structure	QE (%) at Bias	Other Performances
electrons tunneling	SJ	1994 [43]	Glass/Au/Me-PTC/Au	1.0 × 10^6^ (*IQE*@600 nm), −16 V	Working temperature: −50 °C
		1996 [47]	ITO/NTCDA/Au	1.3 × 10^7^ (*IQE*@400 nm), −16 V	Rise time: >60 s
		2000 [26]	ITO/PhEt-PTC/NTCDA/Au	1.7 × 10^7^ (*IQE*@400 nm), −20 V	Rise time: 3.7 s
		2007 [49]	ITO/PEDOT:PSS/C_60_/BCP/Al	5.0 × 10^3^ (*EQE*@450 nm), −4 V	-
		2014 [57]	ITO/PEDOT:PSS/C-TPD:ZnO/C_60_/BCP/Al	4.0 × 10^2^ (*EQE*@390 nm), 8 V	LDR: 120 dB; D*: 3.6 × 10^11^ Jones
	BJ	2002 [51]	ITO/CuPc:C_60_/Au	1.5 × 10^3^ (*IQE*@560 nm), −14 V	Response time: ms
		2010 [52]	ITO/NTCDA/C_60_/CuPc:C_60_/BCP/Al	3.4 × 10^4^ (*EQE*), −4 V	Response time: ms
		2016 [28]	ITO/TPBi/C_70_/TAPC:C_70_/BCP/Al	1.0 × 10^3^ (*EQE*), −4 V	-
			4P-NPB/glass/ITO/TPBi/C_70_/SnPc:C_70_/BCP/Al	1.0 × 10^4^ (*EQE*@780 nm), −10 V	*R*: 70 A/W; D*: 4 × 10^12^ Jones
holes tunneling	SJ	1996 [50]	ITO/DQ/Ag (or Mg)	Ag: 2.5 × 10^5^ (*IQE*@600 nm), 20 V Mg: 1.0 × 10^5^ (*IQE*@600 nm), 36 V	Response time: 10–20 s

**Table 2:** Representative organic polymer PM photodetectors and their performances.

BHJ Type	Year [Ref]	Device	EQE (%) at Bias	Other Performances
Polymer/Organic 1:1	2010 [60]	ITO/PEDOT:PSS/P3HT:PCBM: Ir-125 (1:1:1)/Ca/Al	7.6 × 10^2^@800 nm, −5 V	*R*: 4.9 A/W; Broadband response
	2012 [66]	ITO/PEDOT:PSS/P3HT:PCBM:Q-Switch1 (1:1:1)/Ca/Al	8.4 × 10^2^@560 nm, −5 V	*R*: 4 A/W; Broadband response
		ITO/PEDOT:PSS/P3HT:PCBM: Ir-125:Q-Switch1 (1:1:0.5:0.5)/Ca/Al	5.5 × 10^3^@560 nm, −3.7 V	*R*: 23 A/W; Broadband response
	2014 [61]	ITO/s-Au/P3HT:PCBM (1:1)/Al	1.5 × 10^3^@400 nm, −2 V	-
	2017 [67]	ITO+PEIE/P3HT:PC_61_BM (1:1)/Al	3.3 × 10^3^@370 nm, −1 V	*R*: 14.2 A/W; D*: 1.0 × 10^12^ Jones; Time: 78 µs (rise), 87 µs (decay)
Polymer/Organic Higher than 1:1	2015 [62]	ITO/PEDOT:PSS/P3HT:PC_71_BM (100:1)/LiF/Al	1.7 × 10^4^@380 nm, −19 V	-
	2015 [79]	ITO/PEDOT:PSS/P3HT:PTB7-Th: PC_71_BM (50:50:1)/Al	3.8 × 10^4^@750 nm, −25 V	*R*: 229.5 A/W; D*:1.9 × 10^13^ Jones; Broadband response
	2016 [73]	ITO/PFN/P3HT:ITIC (100:1)/Al	2.3 × 10^3^@625 nm, −15 V	*R*: 41.9 A/W; D*: 7.1 × 10^12^ Jones
	2017 [91,92]	ITO/PFN-OX/P3HT:PC_61_BM (100:1, 4 μm)/Al	8.2 × 10^3^@650 nm, 60 V	D*: 7.7 × 10^11^ Jones@10 V; Narrowband response
	2017 [74]	ITO/PEDOT:PSS/F8T2:PC_71_BM (100:4)/LiF/Al	5.6 × 10^3^@360 nm, −40 V	*R*: 15.9 A/W
	2018 [93]	ITO/PFN-OX/P3HT:PTB7-Th: PC_61_BM (40:60:1,3 μm)/Al	2.0 × 10^2^@800 nm, −50 V	D*: >1.0 × 10^11^ Jones@10 V; LDR: 180 dB@550 nm, 30 V; Narrowband response
Polymer/Organic Lower than 1:1	2016 [64]	ITO/ZnO/PDPP3T:PC_71_BM (1:2)/Al	1.4 × 10^5^@680 nm, 0.5 V	D*: 6.3 × 10^12^ Jones; Decay time:0.27 s
	2017 [75]	ITO//Lys/ PBDTT-PP:PC_71_BM (1:2)/MoO_3_/Al	5.0 × 10^3^@730 nm, 1 V	*R*: 29.5 A/W; D*: 1.6 × 10^15^ Jones @735 nm; Time: 162 μs (rise), 7.9 ms (decay); LDR: 160 dB
Polymer/Inorganic	2008 [62]	ITO/PEDOT:PSS/P3HT:PCBM:CdTe (1:1)/Ca/Al	8.0 × 10^4^@350 nm, −9 V	-
	2012 [8]	ITO/PEDOT:PSS/PVK:TPD-Si_2_/P3HT:ZnO/BCP/Al	3.4 × 10^5^@360 nm, −9 V	*R*: 1001 A/W; D*: 3.4 × 10^15^ Jones; Time: 25 µs (rise), 142 µs (decay)
	2015 [76]	ITO/PEDOT:PSS/PVK:TPD-Si_2_/PDTP-FBT:ZnONPs (1:3)/BCP/Al	2.5 × 10^2^@800 nm, −4.5 V	*R*: 1.6 A/W; D*: 7.1 × 10^9^ Jones
	2016 [95]	ITO/SnO_2_/PEIE/PDTP-DFBT:PC_71_BM:PbS QDs (4 μm)/MoO_3_/Ag	1.8 × 10^2^@890 nm, −7 V	*R*: 1.3 A/W; D*: 8.0 × 10^11^ Jones; LDR: 110 dB; Time: 318 μs; Narrowband response
	2016 [87]	ITO/PVK/P3HT:PC_60_BM:CdTe QDs (1:1, 3.5 μm)/BCP/Al	2.0 × 10^2^@660 nm, −6 V	D*: 7.3 × 10^11^ Jones; LDR: 110 dB; Narrowband response
Polymer/Insulator	2015 [78]	Au/Y-TiOPc@PC/Au	3.6 × 10^4^@830 nm, 225 kV/cm	LDR: 7.1 dB@808 nm
	2016 [27]	ITO/Y-TiOPc NPs/m-TPD/Al	3.5 × 10^5^@780 nm, 15 V/µm	*R*: 2227 A/W; D*: 3.1 × 10^14^ Jones; Broadband response

## References

[1] Rogalski A., Antoszewski J., Faraone L. (2009). Third-generation infrared photodetector arrays. J. Appl. Phys..

[2] Kim S., Lim Y.T., Soltesz E.G., De Grand A.M., Lee J., Nakayama A., Parker J.A., Mihaljevic T., Laurence R.G., Dor D.M. (2003). Near-infrared fluorescent type II quantum dots for sentinel lymph node mapping. Nat. Biotechnol..

[3] Sukhovatkin V., Hinds S., Brzozowski L., Sargent E.H. (2009). Colloidal Quantum-Dot Photodetectors Exploiting Multiexciton Generation. Science.

[4] Gong X., Tong M., Xia Y., Cai W., Moon J.S., Cao Y., Yu G., Shieh C.-L., Nilsson B., Heeger A.J. (2009). High-Detectivity Polymer Photodetectors with Spectral Response from 300 nm to 1450 nm. Science.

[5] Li W.-D., Chou S.Y. (2010). Solar-blind deep-UV band-pass filter (250–350 nm) consisting of a metal nano-grid fabricated by nanoimprint lithography. Opt. Express.

[6] McDonald S.A., Konstantatos G., Zhang S., Cyr P.W., Klem E.J., Levina L., Sargent E.H. (2005). Solution-processed PbS quantum dot infrared photodetectors and photovoltaics. Nat. Mater..

[7] Dou L., Yang Y.M., You J., Hong Z., Chang W.H., Li G., Yang Y. (2014). Solution-processed hybrid perovskite photodetectors with high detectivity. Nat. Commun..

[8] Guo F., Yang B., Yuan Y., Xiao Z., Dong Q., Bi Y., Huang J. (2012). A nanocomposite ultraviolet photodetector based on interfacial trap-controlled charge injection. Nat. Nanotechnol..

[9] Konstantatos G., Sargent E.H. (2010). Nanostructured materials for photon detection. Nat. Nanotechnol..

[10] Wang X., Cheng Z., Xu K., Tsang H.K., Xu J.-B. (2013). High-responsivity graphene/silicon-heterostructure waveguide photodetectors. Nat. Photonics.

[11] Koppens F.H., Mueller T., Avouris P., Ferrari A.C., Vitiello M.S., Polini M. (2014). Photodetectors based on graphene, other two-dimensional materials and hybrid systems. Nat. Nanotechnol..

[12] Liu Z., Parvez K., Li R., Dong R., Feng X., Mullen K. (2015). Transparent conductive electrodes from graphene/PEDOT:PSS hybrid inks for ultrathin organic photodetectors. Adv. Mater..

[13] Kang Y., Liu H.-D., Morse M., Paniccia M.J., Zadka M., Litski S., Sarid G., Pauchard A., Kuo Y.-H., Chen H.-W. (2009). Monolithic germanium/silicon avalanche photodiodes with 340 GHz gain–bandwidth product. Nat. Photonics.

[14] Fang Z., Wang Y., Liu Z., Schlather A., Ajayan P.M., Koppens F.H.L., Nordlander P., Halas N.J. (2012). Plasmon-Induced Doping of Graphene. ACS Nano.

[15] Ju Y., Song J., Geng Z., Zhang H., Wang W., Xie L., Yao W., Li Z. (2012). A microfluidics cytometer for mice anemia detection. Lab Chip.

[16] Kabir M.Z., Hijazi N. (2014). Temperature and field dependent effective hole mobility and impact ionization at extremely high fields in amorphous selenium. Appl. Phys. Lett..

[17] Ribordy G., Gautier J.-D., Zbinden H., Gisin N. (1998). Performance of InGaAs/InP avalanche photodiodes as gated-mode photon counters. Appl. Opt..

[18] Hayden O., Agarwal R., Lieber C.M. (2006). Nanoscale avalanche photodiodes for highly sensitive and spatially resolved photon detection. Nat. Mater..

[19] Cova S., Ghioni M., Lacaita A., Samori C., Zappa F. (1996). Avalanche photodiodes and quenching circuits for single-photon detection. Appl. Opt..

[20] Pearsall T.P., Temkin H., Bean J.C., Luryi S. (1986). Avalanche gain in GexSi1-x/Si infrared waveguide detectors. IEEE Electron Device Lett..

[21] Renker D. (2006). Geiger-mode avalanche photodiodes, history, properties and problems. Nucl. Instrum. Methods Phys. Res. Sect. A.

[22] Reznik A., Zhao W., Ohkawa Y., Tanioka K., Rowlands J.A. (2007). Applications of avalanche multiplication in amorphous selenium to flat panel detectors for medical applications. J. Mater. Sci. Mater. Electron..

[23] Baierl D., Fabel B., Gabos P., Pancheri L., Lugli P., Scarpa G. (2010). Solution-processable inverted organic photodetectors using oxygen plasma treatment. Org. Electron..

[24] Nalwa K.S., Cai Y., Thoeming A.L., Shinar J., Shinar R., Chaudhary S. (2010). Polythiophene-fullerene based photodetectors: Tuning of spectral response and application in photoluminescence based (bio)chemical sensors. Adv. Mater..

[25] Leem D.-S., Lee K.-H., Park K.-B., Lim S.-J., Kim K.-S., Wan Jin Y., Lee S. (2013). Low dark current small molecule organic photodetectors with selective response to green light. Appl. Phys. Lett..

[26] Nakayama K.-I., Hiramoto M., Yokoyama M. (2000). A high-speed photocurrent multiplication device based on an organic double-layered structure. Appl. Phys. Lett..

[27] Li X., Wang S., Xiao Y., Li X. (2016). A trap-assisted ultrasensitive near-infrared organic photomultiple photodetector based on Y-type titanylphthalocyanine nanoparticles. J. Mater. Chem. C.

[28] Yang D., Zhou X., Wang Y., Vadim A., Alshehri S.M., Ahamad T., Ma D. (2016). Deep ultraviolet-to-NIR broad spectral response organic photodetectors with large gain. J. Mater. Chem. C.

[29] Wang X., Li H., Su Z., Fang F., Zhang G., Wang J., Chu B., Fang X., Wei Z., Li B. (2014). Efficient organic near-infrared photodetectors based on lead phthalocyanine/C60 heterojunction. Org. Electron..

[30] Yang D., Zhang L., Yang S.Y., Zou B.S. (2015). Low-voltage pentacene photodetector based on a vertical transistor configuration. Acta Phys. Sin..

[31] Rauch T., Böberl M., Tedde S.F., Fürst J., Kovalenko M.V., Hesser G., Lemmer U., Heiss W., Hayden O. (2009). Near-infrared imaging with quantum-dot-sensitized organic photodiodes. Nat. Photonics.

[32] Peumans P., Bulović V., Forrest S.R. (2000). Efficient, high-bandwidth organic multilayer photodetectors. Appl. Phys. Lett..

[33] Wu S.-H., Li W.-L., Chu B., Su Z.-S., Zhang F., Lee C.S. (2011). High performance small molecule photodetector with broad spectral response range from 200 to 900 nm. Appl. Phys. Lett..

[34] Alvarado S.F., Seidler P.F., Lidzey D.G., Bradley D.D.C. (1998). Direct Determination of the Exciton Binding Energy of Conjugated Polymers Using a Scanning Tunneling Microscope. Phys. Rev. Lett..

[35] Caserta G., Rispoli B., Serra A. (1969). Space-Charge-Limited Current and Band Structure in Amorphous Organic Films. Phys. Status Solidi.

[36] Scharber M.C., Mühlbacher D., Koppe M., Denk P., Waldauf C., Heeger A.J., Brabec C.J. (2006). Design Rules for Donors in Bulk-Heterojunction Solar Cells—Towards 10% Energy-Conversion Efficiency. Adv. Mater..

[37] Xue J., Uchida S., Rand B.P., Forrest S.R. (2004). 4.2% efficient organic photovoltaic cells with low series resistances. Appl. Phys. Lett..

[38] He Z., Zhong C., Su S., Xu M., Wu H., Cao Y. (2012). Enhanced power-conversion efficiency in polymer solar cells using an inverted device structure. Nat. Photonics.

[39] Jansen-van Vuuren R.D., Armin A., Pandey A.K., Burn P.L., Meredith P. (2016). Organic Photodiodes: The Future of Full Color Detection and Image Sensing. Adv. Mater..

[40] Streetman B.G., Banerjee S. (2005). Solid-State Electronic Devices.

[41] Sze S.M., Ng K.K. (2006). Physics of Semiconductor Devices.

[42] Ahmadi M., Wu T., Hu B. (2017). A Review on Organic–Inorganic Halide Perovskite Photodetectors: Device Engineering and Fundamental Physics. Adv. Mater..

[43] Hiramoto M., Imahigashi T., Yokoyama M. (1994). Photocurrent multiplication in organic pigment films. Appl. Phys. Lett..

[44] Hiramoto M., Nakayama K.-I., Katsume T., Yokoyama M. (1998). Field-activated structural traps at organic pigment/metal interfaces causing photocurrent multiplication phenomena. Appl. Phys. Lett..

[45] Hiramoto M., Nakayama K., Sato I., Kumaoka H., Yokoyama M. (1998). Photocurrent multiplication phenomena at organic/metal and organic/organic interfaces. Thin Solid Films.

[46] Nakayama K.-I., Hiramoto M., Yokoyama M. (2000). Photocurrent multiplication at organic/metal interface and surface morphology of organic films. J. Appl. Phys..

[47] Katsume T., Hiramoto M., Yokoyama M. (1996). Photocurrent multiplication in naphthalene tetracarboxylic anhydride film at room temperature. Appl. Phys. Lett..

[48] Hiramoto M., Miki A., Yoshida M., Yokoyama M. (2002). Photocurrent multiplication in organic single crystals. Appl. Phys. Lett..

[49] Huang J., Yang Y. (2007). Origin of photomultiplication in C_60_ based devices. Appl. Phys. Lett..

[50] Hiramoto M., Kawase S., Yokoyama M. (1996). Photoinduced Hole Injection Multiplication in p-Type Quinacridone Pigment Films. Jpn. J. Appl. Phys..

[51] Matsunobu G., Oishi Y., Yokoyama M., Hiramoto M. (2002). High-speed multiplication-type photodetecting device using organic codeposited films. Appl. Phys. Lett..

[52] Hammond W.T., Xue J. (2010). Organic heterojunction photodiodes exhibiting low voltage, imaging-speed photocurrent gain. Appl. Phys. Lett..

[53] Hiramoto M., Fujino K., Yoshida M., Yokoyama M. (2003). Influence of Oxygen and Water on Photocurrent Multiplication in Organic Semiconductor Films. Jpn. J. Appl. Phys..

[54] Hiramoto M., Suemori K., Yokoyama M. (2003). Influence of Oxygen on Photocurrent Multiplication Phenomenon at Organic/Metal Interface. Jpn. J. Appl. Phys..

[55] Hiramoto M., Sato I., Nakayama K.-I., Yokoyama M. (1998). Photocurrent multiplication at organic/metal interface and morphology of metal films. Jpn. J. Appl. Phys..

[56] Guo F., Xiao Z., Huang J. (2013). Fullerene Photodetectors with a Linear Dynamic Range of 90 dB Enabled by a Cross-Linkable Buffer Layer. Adv. Opt. Mater..

[57] Fang Y., Guo F., Xiao Z., Huang J. (2014). Large Gain, Low Noise Nanocomposite Ultraviolet Photodetectors with a Linear Dynamic Range of 120 dB. Adv. Opt. Mater..

[58] Däubler T.K., Neher D., Rost H., Hörhold H.H. (1999). Efficient bulk photogeneration of charge carriers and photoconductivity gain in arylamino-PPV polymer sandwich cells. Phys. Rev. B Condens. Matter.

[59] Campbell I.H., Crone B.K. (2007). Bulk photoconductive gain in poly(phenylene vinylene) based diodes. J. Appl. Phys..

[60] Chen F.-C., Chien S.-C., Cious G.-L. (2010). Highly sensitive, low-voltage, organic photomultiple photodetectors exhibiting broadband response. Appl. Phys. Lett..

[61] Melancon J.M., Živanović S.R. (2014). Broadband gain in poly(3-hexylthiophene):phenyl-C_61_-butyric-acid-methyl-ester photodetectors enabled by a semicontinuous gold interlayer. Appl. Phys. Lett..

[62] Li L., Zhang F., Wang J., An Q., Sun Q., Wang W., Zhang J., Teng F. (2015). Achieving EQE of 16,700% in P3HT:PC_71_BM based photodetectors by trap-assisted photomultiplication. Sci. Rep..

[63] Li L., Zhang F., Wang W., Fang Y., Huang J. (2015). Revealing the working mechanism of polymer photodetectors with ultra-high external quantum efficiency. Phys. Chem. Chem. Phys..

[64] Zhou X., Yang D., Ma D., Vadim A., Ahamad T., Alshehri S.M. (2016). Ultrahigh Gain Polymer Photodetectors with Spectral Response from UV to Near-Infrared Using ZnO Nanoparticles as Anode Interfacial Layer. Adv. Funct. Mater..

[65] Chen H.Y., Lo M.K., Yang G., Monbouquette H.G., Yang Y. (2008). Nanoparticle-assisted high photoconductive gain in composites of polymer and fullerene. Nat. Nanotechnol..

[66] Chuang S.-T., Chien S.-C., Chen F.-C. (2012). Extended spectral response in organic photomultiple photodetectors using multiple near-infrared dopants. Appl. Phys. Lett..

[67] Wang T., Hu Y., Deng Z., Wang Y., Lv L., Zhu L., Lou Z., Hou Y., Teng F. (2017). High sensitivity, fast response and low operating voltage organic photodetectors by incorporating a water/alcohol soluble conjugated polymer anode buffer layer. RSC Adv..

[68] Wang Y., Zhu L., Hu Y., Deng Z., Lou Z., Hou Y., Teng F. (2017). High sensitivity and fast response solution processed polymer photodetectors with polyethylenimine ethoxylated (PEIE) modified ITO electrode. Opt. Express.

[69] Li L., Zhang F., Wang W., An Q., Wang J., Sun Q., Zhang M. (2015). Trap-assisted photomultiplication polymer photodetectors obtaining an external quantum efficiency of 37,500%. ACS Appl. Mater. Interfaces.

[70] Wang W., Zhang F., Li L., Gao M., Hu B. (2015). Improved Performance of Photomultiplication Polymer Photodetectors by Adjustment of P3HT Molecular Arrangement. ACS Appl. Mater. Interfaces.

[71] Han Z., Zhang H., Tian Q., Li L., Zhang F. (2015). Solution-processed polymer photodetectors with trap-assisted photomultiplication. Sci. China Phys. Mech..

[72] Wang W., Zhang F., Bai H., Li L., Gao M., Zhang M., Zhan X. (2016). Photomultiplication photodetectors with P3HT:fullerene-free material as the active layers exhibiting a broad response. Nanoscale.

[73] Miao J., Zhang F., Lin Y., Wang W., Gao M., Li L., Zhang J., Zhan X. (2016). Highly Sensitive Organic Photodetectors with Tunable Spectral Response under Bi-Directional Bias. Adv. Opt. Mater..

[74] Esopi M.R., Calcagno M., Yu Q. (2017). Organic Ultraviolet Photodetectors Exhibiting Photomultiplication, Low Dark Current, and High Stability. Adv. Mater. Technol..

[75] Nie R., Deng X., Feng L., Hu G., Wang Y., Yu G., Xu J. (2017). Highly Sensitive and Broadband Organic Photodetectors with Fast Speed Gain and Large Linear Dynamic Range at Low Forward Bias. Small.

[76] Shen L., Fang Y., Dong Q., Xiao Z., Huang J. (2015). Improving the sensitivity of a near-infrared nanocomposite photodetector by enhancing trap induced hole injection. Appl. Phys. Lett..

[77] Dong R., Bi C., Dong Q., Guo F., Yuan Y., Fang Y., Xiao Z., Huang J. (2014). An Ultraviolet-to-NIR Broad Spectral Nanocomposite Photodetector with Gain. Adv. Opt. Mater..

[78] Peng W., Liu Y., Wang C., Hu R., Zhang J., Xu D., Wang Y. (2015). A highly sensitive near-infrared organic photodetector based on oxotitanium phthalocyanine nanocrystals and light-induced enhancement of electron tunnelling. J. Mater. Chem. C.

[79] Wang W., Zhang F., Li L., Zhang M., An Q., Wang J., Sun Q. (2015). Highly sensitive polymer photodetectors with a broad spectral response range from UV light to the near infrared region. J. Mater. Chem. C.

[80] Gao M., Wenbin W., Li L., Miao J., Zhang F. (2017). Highly sensitive polymer photodetectors with a wide spectral response range. Chin. Phys. B.

[81] Ameri T., Khoram P., Min J., Brabec C.J. (2013). Organic ternary solar cells: A review. Adv. Mater..

[82] An Q., Zhang F., Gao W., Sun Q., Zhang M., Yang C., Zhang J. (2018). High-efficiency and air stable fullerene-free ternary organic solar cells. Nano Energy.

[83] An Q., Zhang F., Li L., Wang J., Zhang J., Zhou L., Tang W. (2014). Improved efficiency of bulk heterojunction polymer solar cells by doping low-bandgap small molecules. ACS Appl. Mater. Interfaces.

[84] An Q., Zhang F., Sun Q., Zhang M., Zhang J., Tang W., Yin X., Deng Z. (2016). Efficient organic ternary solar cells with the third component as energy acceptor. Nano Energy.

[85] Kokil A., Poe A.M., Bae Y., Della Pelle A.M., Homnick P.J., Lahti P.M., Kumar J., Thayumanavan S. (2014). Improved performances in polymer BHJ solar cells through frontier orbital tuning of small molecule additives in ternary blends. ACS Appl. Mater. Interfaces.

[86] Dandin M., Abshire P., Smela E. (2007). Optical filtering technologies for integrated fluorescence sensors. Lab Chip.

[87] Olbright G.R., Peyghambarian N., Gibbs H.M., Macleod H.A., Van Milligen F. (1984). Microsecond room-temperature optical bistability and crosstalk studies in ZnS and ZnSe interference filters with visible light and milliwatt powers. Appl. Phys. Lett..

[88] Armin A., Jansen-van Vuuren R.D., Kopidakis N., Burn P.L., Meredith P. (2015). Narrowband light detection via internal quantum efficiency manipulation of organic photodiodes. Nat. Commun..

[89] Li Z., Butun S., Aydin K. (2015). Large-Area, Lithography-Free Super Absorbers and Color Filters at Visible Frequencies Using Ultrathin Metallic Films. ACS Photonics.

[90] Xu T., Wu Y.K., Luo X., Guo L.J. (2010). Plasmonic nanoresonators for high-resolution colour filtering and spectral imaging. Nat. Commun..

[91] Wang W., Zhang F., Du M., Li L., Zhang M., Wang K., Wang Y., Hu B., Fang Y., Huang J. (2017). Highly Narrowband Photomultiplication Type Organic Photodetectors. Nano Lett..

[92] Miao J., Zhang F., Du M., Wang W., Fang Y. (2017). Photomultiplication type narrowband organic photodetectors working at forward and reverse bias. Phys. Chem. Chem. Phys..

[93] Miao J., Zhang F., Du M., Wang W., Fang Y. (2018). Photomultiplication Type Organic Photodetectors with Broadband and Narrowband Response Ability. Adv. Opt. Mater..

[94] Shen L., Fang Y., Wei H., Yuan Y., Huang J. (2016). A Highly Sensitive Narrowband Nanocomposite Photodetector with Gain. Adv. Mater..

[95] Shen L., Zhang Y., Bai Y., Zheng X., Wang Q., Huang J. (2016). A filterless, visible-blind, narrow-band, and near-infrared photodetector with a gain. Nanoscale.

[96] Reynaert J., Arkhipov V.I., Heremans P., Poortmans J. (2006). Photomultiplication in Disordered Unipolar Organic Materials. Adv. Funct. Mater..

[97] Campbell I.H., Crone B.K. (2009). A near infrared organic photodiode with gain at low bias voltage. Appl. Phys. Lett..

[98] Qi D., Fischbein M., Drndić M., Šelmić S. (2005). Efficient polymer-nanocrystal quantum-dot photodetectors. Appl. Phys. Lett..

[99] Cui Y., Fung K.H., Xu J., Ma H., Jin Y., He S., Fang N.X. (2012). Ultrabroadband light absorption by a sawtooth anisotropic metamaterial slab. Nano Lett..

[100] Cui Y., He Y., Jin Y., Ding F., Yang L., Ye Y., Zhong S., Lin Y., He S. (2014). Plasmonic and metamaterial structures as electromagnetic absorbers. Laser Photonics Rev..

[101] Wang W., Hao Y., Cui Y., Tian X., Zhang Y., Wang H., Shi F., Wei B., Huang W. (2014). High-efficiency, broad-band and wide-angle optical absorption in ultra-thin organic photovoltaic devices. Opt. Express.

[102] Cui Y., Zhao H., Yang F., Tong P., Hao Y., Sun Q., Shi F., Zhan Q., Wang H., Zhu F. (2015). Efficiency enhancement in organic solar cells by incorporating silica-coated gold nanorods at the buffer/active interface. J. Mater. Chem. C.

[103] Hao Y., Song J., Yang F., Hao Y., Sun Q., Guo J., Cui Y., Wang H., Zhu F. (2015). Improved performance of organic solar cells by incorporating silica-coated silver nanoparticles in the buffer layer. J. Mater. Chem. C.

[104] Wang Z., Hao Y., Wang W., Cui Y., Sun Q., Ji T., Li Z., Wang H., Zhu F. (2016). Incorporating silver-SiO_2_ core-shell nanocubes for simultaneous broadband absorption and charge collection enhancements in organic solar cells. Synth. Met..

[105] Ji T., Wang Y., Cui Y., Lin Y., Hao Y., Li D. (2017). Flexible broadband plasmonic absorber on moth-eye substrate. Mater. Today Energy.

[106] Liu D., Liang Q., Li G., Gao X., Wang W., Zhan Q., Ji T., Hao Y., Cui Y. (2017). Improved efficiency of organic photovoltaic cells by incorporation of auag-alloyed nanoprisms. IEEE J. Photovolt..

[107] Wang W., Cui Y., Fung K.H., Zhang Y., Ji T., Hao Y. (2017). Comparison of Nanohole-Type and Nanopillar-Type Patterned Metallic Electrodes Incorporated in Organic Solar Cells. Nanoscale Res. Lett..

[108] Peter Amalathas A., Alkaisi M.M. (2017). Efficient light trapping nanopyramid structures for solar cells patterned using UV nanoimprint lithography. Mater. Sci. Semicond. Process..

[109] Zhang C., Song Y., Wang M., Yin M., Zhu X., Tian L., Wang H., Chen X., Fan Z., Lu L. (2017). Efficient and Flexible Thin Film Amorphous Silicon Solar Cells on Nanotextured Polymer Substrate Using Sol-gel Based Nanoimprinting Method. Adv. Funct. Mater..

[110] Lin Y., Xu Z., Yu D., Lu L., Yin M., Tavakoli M.M., Chen X., Hao Y., Fan Z., Cui Y. (2016). Dual-Layer Nanostructured Flexible Thin-Film Amorphous Silicon Solar Cells with Enhanced Light Harvesting and Photoelectric Conversion Efficiency. ACS Appl. Mater. Interfaces.

[111] Zhang Y., Cui Y., Wang W., Fung K.H., Ji T., Hao Y., Zhu F. (2014). Absorption Enhancement in Organic Solar Cells with a Built-In Short-Pitch Plasmonic Grating. Plasmonics.

[112] Rui D., Yanjun F., Jungseok C., Jun D., Zhengguo X., Qingfeng D., Yongbo Y., Andrea C., Cheng Z.X., Jinsong H. (2015). High-Gain and Low-Driving-Voltage Photodetectors Based on Organolead Triiodide Perovskites. Adv. Mater..

[113] Liu C., Peng H., Wang K., Wei C., Wang Z., Gong X. (2016). PbS quantum dots-induced trap-assisted charge injection in perovskite photodetectors. Nano Energy.

[114] Chen H.-W., Sakai N., Jena A.K., Sanehira Y., Ikegami M., Ho K.-C., Miyasaka T. (2015). A Switchable High-Sensitivity Photodetecting and Photovoltaic Device with Perovskite Absorber. J. Phys. Chem. Lett..

[115] Konrad D., Wolfgang T., Thomas M., Michael S., Khaja N.M., Michael G. (2015). Working Principles of Perovskite Photodetectors: Analyzing the Interplay Between Photoconductivity and Voltage-Driven Energy-Level Alignment. Adv. Funct. Mater..

[116] Goh C., Scully S.R., McGehee M.D. (2007). Effects of molecular interface modification in hybrid organic-inorganic photovoltaic cells. J. Appl. Phys..

[117] Levell J.W., Giardini M.E., Samuel I.D.W. (2010). A hybrid organic semiconductor/silicon photodiode for efficient ultraviolet photodetection. Opt. Express.

[118] Yakuphanoglu F. (2007). Photovoltaic properties of hybrid organic/inorganic semiconductor photodiode. Synth. Met..

[119] Jariwala D., Marks T.J., Hersam M.C. (2017). Mixed-dimensional van der Waals heterostructures. Nat. Mater..

[120] Jariwala D., Howell S.L., Chen K.S., Kang J., Sangwan V.K., Filippone S.A., Turrisi R., Marks T.J., Lauhon L.J., Hersam M.C. (2016). Hybrid, Gate-Tunable, van der Waals p-n Heterojunctions from Pentacene and MoS2. Nano Lett..

[121] Jariwala D., Sangwan V.K., Wu C.-C., Prabhumirashi P.L., Geier M.L., Marks T.J., Lauhon L.J., Hersam M.C. (2013). Gate-tunable carbon nanotube–MoS^2^ heterojunction p-n diode. Proc. Natl. Acad. Sci. USA.

[122] Wang J.-J., Wang Y.-Q., Cao F.-F., Guo Y.-G., Wan L.-J. (2010). Synthesis of Monodispersed Wurtzite Structure CuInSe2 Nanocrystals and Their Application in High-Performance Organic−Inorganic Hybrid Photodetectors. J. Am. Chem. Soc..

